# Impact of sports activity on Polish adults: Self-reported health, social capital & attitudes

**DOI:** 10.1371/journal.pone.0226812

**Published:** 2019-12-19

**Authors:** Łukasz Skrok, Dawid Majcherek, Hanna Nałęcz, Elżbieta Biernat

**Affiliations:** 1 Department of Business Economics, Collegium of World Economy, SGH Warsaw School of Economics, al. Niepodległości, Warsaw, Poland; 2 Collegium of World Economy, SGH Warsaw School of Economics, al. Niepodległości, Warsaw, Poland; 3 Department of Children and Adolescents Health, Institute of Mother and Child, Warsaw, Poland; 4 Department of Tourism, Collegium of World Economy, SGH Warsaw School of Economics, al. Niepodległości, Warsaw, Poland; University of Jyvaskyla, FINLAND

## Abstract

The aim of our study was a quasi-experimental estimation of the relationship between sports activity and social capital, and an assessment made at an individual level of the impact of sports activity on health, beliefs and the social situation of Polish adults. Matching estimation method and Social Diagnosis data were used. The dataset enables not only an analysis of the self-reported health, but also of social capital. The panel structure of the data and the applied method allow for stratifying the sample using past characteristics, including past sports activity, as well as for assessing outcomes after the treatment—engaging in sports activity—takes place. Both allow for an interpretation of the results as reflections of a causal relationship. Even though publications applying matching estimation or IV estimation to assess the impact of sports activity have recently been increasing in number, ours is the first to concentrate on the effects on multidimensional social capital for adults using panel data and matching estimation. It is also the first to focus on a country that is neither one of the most developed in the world or one of the least developed. The results obtained suggest significant heterogeneity in terms of age, gender and place of living. We also found that sports contributed to building social networks and being socially active. Our research indicates that sports activity enhances social health. It can be used as a tool for a policy of social activation and strengthening the health potential of adults, especially those over 40.

## Introduction

Social capital (SC)—defined by the classic authors Bourdieu [1] and Putnam [2], as well as the World Bank and OECD [3] − is not a precisely defined asset, or directly measurable feature, such as an inclination to trust other people. It is more a collection of various factors shaping interpersonal relations [4]. The OECD definition specifies SC as "networks together with shared norms, values and understandings that facilitate cooperation within or among groups". It indicates that having educated, healthy and happy citizens is not enough for a country’s economic development. Additional instruments are needed to trigger the synergy effect of different capitals in a social dimension [5]. In the case of health as one of the elements of human capital, and in a wider sense social capital [6], it is important to cooperate with different dimensions of the social environment. McNeill et al. [7] distinguish the following:

- social support and social networks,
- socio-economic position,
- racial discrimination,
- factors related to the local environment (neighbourhood),
- social cohesion and social capital.

Synergy of the other listed dimensions supports the building of SC. An individual’s attitude and relatively stable organization of beliefs (as regards an object or situation) play an important role, predisposing people to respond to the above mentioned in a self-specific way [8]. Attitudes and behaviours towards health are not only the personal achievements of the individual, but also the result of the powerful influence of a combination of all factors (including customs and beliefs) [9]. Lewicki [10] classifies health attitudes as: ideological, social, interpersonal, intrapersonal, cultural, intellectual, as well as the results of human relations with nature. Recognizing them is one of the ways to understand and consciously explain the pro and anti-health consequences towards self, others and the environment.

Over the last 20 years, SC research has been conducted in the context of health, in parallel with research on the general concept of SC [11]. The beginning of a special interest in health within the context of social relations began in the 90s and is still growing. According to Islam et al. [12], in the years 1995–2005, 42 works in this field were published. It is worth noting that there have already been analyses regarding the influence of some elements of SC on people’s health, i.e. suicides [13]. Following the previous reports, only selected elements of SC correlate with health most often, and there is no clear relationship between SC and health [14]. Some of the works provide evidence for a relationship between SC and good health in the area of voluntary activity, networks of friendship, religious commitment, and participation in hobby clubs [15]. Others show that a low level of SC affects health negatively. Nyqvist has made a summary of the research carried out in various countries in the years 2000–2007 [16]. In her report, health is most often positively correlated with trust, strong relationships with close people, implementation of reciprocity in interpersonal relations, social involvement, commitment to religious organizations, and spending leisure time in hobby associations. Perceiving social support, participation in voluntary work, the ability to rely on friends during illness, and a sense of safety also have a positive impact on health [16].

Physical activity is an important determinant of health. The results of epidemiological and clinical studies show that lack of physical activity is the cause of at least 20 chronic diseases. Hypokinesis—lack of physical activity, is considered one of the sources of the obesity epidemic [17], and comorbid metabolic syndrome [18], as well as one of the causes of ischemic heart disease [19], and cancer [20], and is responsible for 6% of deaths worldwide [21]. Also, the connection between physical activity (PA)/sport activity (SA) and SC dimensions is well documented for different countries [22, 23, 24, 25]. However, little empirical evidence has been found for the causal relationship between participation in sports and SC, with the opposite relationship assessed as much stronger [26]. Felfe et al. [27], in their study of German children, showed the positive impact of participation in sports groups on building a network of friends. It was related to better mental health and the proper social functioning of children. Similar conclusions derived from studies on Peruvian children were reached by Pawlowski et al. [28]. They showed the positive effect of participation in sport on children’s development (participation in a sports group had a positive effect both on the self-assessment of their health and selected measures of SC). Nevertheless, research aiming to estimate the causal impact of sports participation on SC has been scarce. For this reason, we have decided to analyze the issue of the relationship between SA and SC using Social Diagnosis, a Polish dataset. The motivation can be traced back to an observation made by the National Bank of Poland [29] in its research on innovation. It observed that out of different measures of SC the one that most clearly shows the difference between Poland and highly innovative countries (which are, in general, the most developed ones) is membership of sports clubs. Therefore, the first aim of our study was a quasi-experimental estimation of the relationship between the SA and the indicators of SC made at an individual level. The second aim was to assess the SA impact on health, beliefs and the social situation of adults.

## Material and method

### Data source

Social Diagnosis (SD) data was used–a dataset based on results of a representative, longitudinal study conducted by an interdisciplinary board of academics in 2000–2015 [30]. Furthermore, the SD dataset has been combined with two other datasets on sports infrastructure: Statistics Poland (SP), conducted every four years, the most recent from 2014, was used for our analysis [31] and the “Orliki” database, containing information on facilities constructed in 2008–2012 within a publicly-funded programme [32]. The data has been combined by calculation indicators of availability: number of sports facilities of particular types per capita, at the NUTS (Classification of Territorial Units for Statistics) 3 level.

To the best of our knowledge, the results of research using these combined datasets have not previously been reported.

Even though the SD survey has been conducted over 15 years, for the purpose of our research only the last three biannual editions were suitable (2011, 2013, and 2015), with 26 453 household members taking part in the individual survey. Of these, 18 020 remained in the 2013 sample and 11 461 in the 2015 sample.

Sports activity participation was measured on the basis of the question ‘Do you practise any sports or physical activity?’ with a cafeteria of answers: (1) aerobics; (2) running/jogging/Nordic walking; (3) gym; (4) cycling; (5) skiing or other winter sports; (6) swimming; (7) football or other team sports; (8) yoga; (9) martial arts; as well as an ‘other’ category and a negative answer. The unweighted ratio of declarations of being active varied between 35–36% in 2011, 2013 and 2015.

Lastly, the term ‘physical activity’ is used in the official translation of the questionnaire on the diagnoza.com webpage. However, the analysis of possible answers suggests that these domains should be understood as SA, which is a narrower term, e.g. does not include walking, and that interpretation was applied in our study.

The majority of variables characterising individuals in the sample are dummy variables, describing declarations of the occurrence of particular events or states in their lives, e.g. involvement in voluntary activities, ailments or retirement; categorical variables, e.g. dissatisfaction with own’s health, measured on a 1–6 point scale, or a lust for life, measured on a 1–10 point scale; as well as continuous variables, e.g. age or number of years in education). Details of the construction and characteristic of the variables in the sample are included in S1 and S2 Appendices.

### Method

The estimation method utilizes a distance-weighted radius matching estimator with bias adjustment proposed by Lechner et al. [33] and its implementation as a Stata module by Huber et al. [34] The pseudo-experimental research design follows recent literature on the effects of PA, sports participation or sports infrastructure, and in particular the one used by Lechner [35], Lechner & Sari [36], Felfe et al. [27], Pawlowski et al. [28], Schüttoff et al. [25]. Furthermore, a part of our dataset and radius matching was previously applied to analyse the impact of retirement on SA in Poland by Biernat et al. [37]. Engagement in SA or PA at a given point in time is chosen as a treatment variable. Therefore, treated and non-treated observations in the sample are matched based on the probability of engaging in SA/PA, usually estimated using a probit or logit model. In other words, conditionally on controlled variables, being active or not, can be perceived as a random event. Furthermore, to allow for differences in determination of SA/PA and their impact on measured outcomes, the sample is divided into gender- and past SA/PA-based strata. The latter shows the importance of the longitudinal structure used in the dataset, allowing past behaviour or long-term characteristics to be taken into account. In this case, variables used to create strata and calculate the probability of receiving treatment are measured in the pre-treatment period. Also, the outcome monitoring period(s) can be designed as taking place after the treatment period, further enhancing causal inference. Furthermore, the applied method and procedure use technical adjustments which counteract the issues of dissimilarities in treated and non-treated sub-samples or overreliance on propensity score—bias adjustment, matching based on propensity score and additional matching variables, selection of common support.

### Research design

The time structure of the research design has been set based on the three last SD surveys:

- 2011: pre-treatment period,
- 2013: treatment period,
- 2015: outcome monitoring period.

Results from 2011 have been used as a basis for sample stratification and the source of explanatory variables in the first-step estimation of probability of sports participation in 2013 for individuals in the sample (the results of which are reported in S3 Appendix). Outcomes for self-reported health, social capital and life attitudes were measured in 2015.

Therefore, it was possible to investigate the effect of, first, ‘initiating SA’ vs remaining inactive (i.e. comparing participants inactive in 2011 and active in 2013 with inactive in both 2011 and 2013), and second, ‘sustaining SA’ vs ‘ceasing SA’ (i.e. comparing participants active in both 2011 and 2013 with active in 2011 but inactive in 2013).

Initially, we also investigated the labour market outcomes, but most of them remain insignificant (which is in line with the previously mentioned literature, mostly indicating positive impact on adults after substantially longer than two years after treatment period).

The stratification has been based on the following characteristics:

- gender (female/male),
- SA in 2011 (active/inactive),
- age group (25-40/41-64),
- education (high edu level/low edu level; having completed more than 12 years of education and having completed 12 or less, respectively).

Due to the size of the sub-sample, stratification based on age group and education has been used alternatively, along with gender and sports participation in 2011.

A further note regarding the education threshold is necessary. The formation of education stratification is related to stages of education in Poland [38]. Based on the most recent educational reforms, which came into force from 1^st^ September 2018, there are two levels below higher education: one is primary school, which lasts eight years, the other is high school, which lasts four or five years at technical college. There have been a few changes in the history of the Polish education system. Before 1999, the education system included eight years of primary school, after which it was possible to apply to a four or five-year (technical) secondary school or a three-year vocational school. From 1999 to 2018 there was one additional level: junior high school, which lasted three years. However, in this system, primary school lasted six years, high school lasted three years and technical college was four years. Despite this, below the level of higher education, the total number of years remained unchanged. Taking into account the past and current rules, as well as the relative prevalence of non-technical high schools lasting four years in the current/old system, the education cut-off was set at the level of 12 years.

The following options were used in the estimation: linear or logit bias correction, matching using Mahalanobis distance and additional variables from the explanatory variables from the first-step estimation, asymptotic standard errors. Average treatment effects were reported separately for particular strata.

### Sample selection

The sample selected for our study is a working-age group of 25–64. Defining the minimum age at 25 means excluding people at an age typical for participation in the higher education system, and, therefore, differing access and obligation to use sports infrastructure. At the other end of the scale, our sample includes young retired people, including early retirees, with the official retirement age in Poland for women at 60 years of age and 65 for men.

Respondents who had not answered a sports participation question in either 2011 or 2013 have been excluded, resulting in a sample consisting of 12 205 participants. Furthermore, observations with missing information on explanatory variables used in the first step of the estimation procedure have also been excluded.

The final sub-sample consisted of 10 861 respondents, 56% of whom were women, 32% engaged in sports activity in 2011 and 33% in 2013, 67% were over 40 years old, and 61% participated in the education system for up to 12 years.

This study was ethically approved by the National Science Centre, Poland as project No.2017/27/B/HS4/00427 “The role of sports activity in building social capital in Poland”.

### Robustness check

Following the literature on using radius matching to analyse the impact of sports [36, 28], we conduct the robustness check in several ways. First, we check whether sample attrition between 2013 and 2015 is significantly influenced by treatment in 2013. Second, we repeat the estimation for different stratifications (based on marital status and place of living). Third, we report the results of re-estimation for the most interesting outcomes conducted with different technical assumptions. These additional results are included in S4–S6 Appendices.

## Results

The most consistent and robust outcome is the persistence of SA. For most of the strata, individuals active in 2013 were twice or three times as likely to remain so in 2015 compared to non-active ones in 2013.

For example, women aged 41–64, non-active in 2011, the probability of remaining active in 2015 after engaging in SA in 2013 (initiating SA) was 0.452, which was higher by 0.302 than the probability of being active in 2015 while remaining inactive in 2013 (p < 0.001; Table 1).

**Table 1:** Effects of sports activity (‘treatment’) on women belonging to different age groups.

Outcome variable	Age 41–64 non-active women (n = 1,885)		Age 41–64 active women (n = 708)		Age 25–40 non-active women (n = 727)		Age 25–40 active women (n = 593)	
	treatment group (n = 355)	difference	treatment group (n = 401)	Difference	treatment group (n = 155)	difference	treatment group (n = 148)	difference
*Sport participation*								
Sport 2015	0.452	0.302***	0.734	0.331***	0.502	0.242***	0.708	0.284***
>1 category of sport activity 2015	0.061	0.044***	0.248	0.150***	0.171	0.113***	0.382	0.239***
*Health and physical conditions*								
BMI 2015	27.466	0.050	26.734	-0.226	25.129	0.400	24.212	0.260
Dissatisfaction with health 2015	3.176	-0.169**	3.061	-0.025	2.455	-0.325***	2.465	-0.142
Physical problems 2015	0.802	0.004	0.757	0.005	0.395	-0.115**	0.469	-0.038
Health problems 2015	0.681	-0.026	0.680	-0.022	0.431	-0.036	0.410	-0.085
Seriously ill 2014	0.141	-0.016	0.091	-0.045*	0.065	-0.010	0.071	0.000
Tiredness not-related to work 2015	0.450	-0.080***	0.488	0.009	0.279	-0.117***	0.379	0.030
Too much alcohol 2014	0.016	-0.003	0.022	-0.010	0.063	0.042***	0.033	0.011
*Life attitiudes*								
No decrease in energy to work 2015	0.642	0.135***	0.703	0.091**	0.835	0.067*	0.796	0.057
Entire life delightful or pleasing 2015	0.399	0.043	0.481	0.015	0.607	0.152***	0.533	0.084
Lust for life 2015	8.775	0.289***	8.815	0.163	8.770	0.061	8.822	0.073
Achieving goals > fun 2015	0.660	0.010	0.662	-0.026	0.656	0.022	0.642	-0.006
Success depended on her/himself 2015	0.705	0.036	0.719	-0.016	0.762	0.030	0.748	-0.035
Fun is the most important thing 2015	0.581	0.017	0.554	0.006	0.617	-0.022	0.548	0.024
*Social activities*								
Member of sports club 2015	0.008	0.007	0.006	0.006**	0.010	0.010	0.008	-0.003
Number of friends 2015	6.901	1.201***	7.209	0.799	5.036	0.011	6.548	0.456
Number of acquaint. met regularly 2015	5.906	0.568	7.190	0.982	5.592	-0.510	8.160	1.638**
Number of friends met regularly 2015	4.987	0.875***	6.095	1.141**	4.520	-0.199	5.985	0.797*
Most people can be trusted 2015	0.177	0.020	0.143	0.025	0.183	0.038	0.148	0.025
Work for local society 2013–2014	0.193	0.081***	0.264	0.041	0.165	0.021	0.236	-0.053
Member of organisations 2015	0.233	0.078***	0.257	0.030	0.125	-0.012	0.184	0.024
Took part in public meeting 2015	0.223	0.062**	0.288	0.020	0.283	0.024	0.267	-0.031
Voluntary activities 2015	0.118	0.062***	0.128	0.035	0.042	-0.007	0.091	0.032
Voted in elections 2014	0.744	0.001	0.853	0.023	0.721	-0.007	0.744	0.051

The table presents the results of matching estimation for women stratified by age and past SA. For each stratum and each outcome variable, two values are reported: value of the outcome variable for the treated group (i.e. active in 2013; left column) and estimated difference between the treated group and the non-treated group (i.e. inactive in 2013; right column). For the latter, significance is denoted using asterisks

*** p < 0.010

** p < 0.050

* p < 0.100. For the sample sizes, only observations on common support are reported (4.8–21% observations were off common support–with the highest for the active under-40).

For women of the same age, but engaged in SA in 2011, the probability of being active in 2015 of those who remained active in 2013 (sustaining SA) was 0.734, which was greater by 0.331 than the probability of becoming active once again after becoming passive in 2013 (‘ceasing SA’; p < 0.001). The effects for women aged 25–40 were similar, but weaker. Furthermore, all sub-groups were significantly more likely to declare more than one category of activity in this year (Table 1). For example, for non-active women over 40 in 2011 the effect was 0.044, meaning an increase of probability of engaging in at least two categories of SA in 2015 (after initiating SA in 2013) to 0.061 (p *=* 0.003), while the effect sustaining SA was 0.150, meaning an increase to 0.248 (p *<* 0.001). For younger women the effects were similar.

The differences between those initiating and sustaining SA can be observed also with reference to health (Table 1). Only for the former were there significant effects on dissatisfaction with health. Women under the age of 40 evaluated health at the level of 2.455 after initiating SA in 2013, better by 0.325 than those who remained non-active in 2013 (p < 0.001). Those aged over 40 evaluated it at 3.176, better by 0.169 than those who remained non-active (p = 0.021). Both age groups also declared tiredness not related to work less often after initiating SA in 2013 (0.279 vs 0.396 for women younger than 40, p = 0.008; 0.450 vs 0.530 for women over 40, p = 0.008). A stronger positive health effect for younger women is also reflected in significantly the lower ratio of declaring physical problems (‘having ailments making it difficult to leave home, climb stars etc.’) after initiating SA in 2013 (0.395 vs 0.510, p *=* 0.014). This group also has a greater propensity to admit to overusing alcohol after starting SA a year earlier (0.063 vs 0.021, p = 0.008).

The positive effects for women of initiating SA in 2013 can be seen in the case of attitude towards life. The probability of declaring no decrease in energy to work is higher both for respondents under 40 (0.835 vs. 0.768; p = 0.053) and those over 40 (0.642 vs. 0.507; p < 0.001). A similar effect is observed for women over 40 sustaining SA in 2013 (0.703 vs. 0.612; p = 0.015). Initiating SA in 2013 meant a greater lust for life for women over 40 (8.775 vs 8.486, p = 0.003) and a greater ratio of women before 40 declaring that their entire life is delightful/pleasing (0.607 vs 0.455; p = 0.001).

When it comes to social activity, the most consistent result is the positive effect of SA on the number of social relations. For women over 40, initiating SA in 2013 meant having a greater declared number of friends, 6.901 vs. 5.700 (p = 0.001). Furthermore, a positive effect in terms of met friends can be observed for women over 40, both those who sustained SA in 2013 (6.095 vs. 4.954; p = 0.011) or those who initiated SA in 2013 (4.987 vs. 4.112; p = 0.003). For younger women, a positive effect of sustaining SA on the number of acquaintances met regularly was observed (8.160 vs. 6.522; p = 0.040).

Furthermore, a positive effect on general social activity can be observed–after initiating SA in 2013, more women worked for the benefit of local society during the two years prior to the SD survey (0.193 vs. 0.112; p < 0.001), took part in public meetings outside work (0.223 vs 0.161; p = 0.015), engaged in voluntary activities (0.118 vs 0.056; p < 0.001) and, on average, they belong to more organisations (0.233 vs 0.155; p = 0.005).

Estimation results obtained using stratification based on number of years in education (Table 2) is, in the case of most outcomes, consistent with the results presented in Table 1.

**Table 2:** Effects of sports activity (‘treatment’) on women with different number of years in education.

Outcome variable	High edu level non-active women (n = 970)		High edu level active women (n = 605)		Low edu level non-active women (n = 1,689)		Low edu level active women (n = 541)	
	treatment group (n = 241)	difference	treatment group (n = 374)	difference	treatment group (n = 275)	difference	treatment group (n = 267)	difference
*Sport participation*								
Sport 2015	0.518	0.260***	0.800	0.344***	0.407	0.274***	0.653	0.291***
>1 category of sport activity 2015	0.131	0.087***	0.339	0.180***	0.064	0.044***	0.214	0.165***
*Health and physical conditions*								
BMI 2015	25.713	-0.426	25.168	-0.068	27.376	0.431	26.614	-0.050
Dissatisfaction with health 2015	2.857	-0.154*	2.718	-0.047	3.143	-0.142*	3.063	-0.064
Physical problems 2015	0.589	-0.048	0.566	-0.009	0.753	-0.020	0.771	0.029
Health problems 2015	0.553	-0.009	0.504	0.005	0.648	-0.042	0.671	-0.016
Seriously ill 2014	0.136	0.032	0.076	-0.019	0.126	-0.023	0.086	-0.076***
Tiredness not-related to work 2015	0.372	-0.129***	0.447	0.038	0.438	-0.046	0.459	0.007
Too much alcohol 2014	0.052	0.031**	0.029	0.011	0.060	0.039***	0.023	-0.003
*Life attitiudes*								
No decrease in energy to work 2015	0.757	0.077**	0.776	0.052	0.634	0.121***	0.670	0.075*
Entire life delightful or pleasing 2015	0.553	0.089**	0.535	0.020	0.391	0.060*	0.446	0.027
Lust for life 2015	8.974	0.198*	8.886	0.060	8.706	0.337***	8.567	-0.086
Achieving goals > fun 2015	0.673	-0.022	0.694	0.043	0.675	0.061*	0.588	-0.042
Success depended on her/himself 2015	0.795	0.039	0.801	-0.011	0.675	0.042	0.702	-0.002
Fun is the most important thing 2015	0.505	0.001	0.475	-0.041	0.644	0.012	0.671	0.011
*Social activities*								
Member of sports club 2015	0.004	0.003	0.008	0.002	0.008	0.008	0.000	-
Number of friends 2015	6.395	0.728*	5.961	0.266	6.451	1.110**	7.701	0.457
Number of acquaint. met regularly 2015	6.373	-0.077	8.222	1.823***	5.255	0.268	6.020	0.165
Number of friends met regularly 2015	5.098	0.374	5.894	1.092***	4.337	0.318	5.446	0.083
Most people can be trusted 2015	0.270	0.116***	0.178	0.007	0.121	-0.031	0.127	0.027
Work for local society 2013–2014	0.177	0.008	0.261	-0.005	0.179	0.084***	0.214	0.018
Member of organisations 2015	0.220	-0.020	0.314	0.115**	0.172	0.077***	0.147	0.000
Took part in public meeting 2015	0.257	0.039	0.302	0.010	0.228	0.066**	0.243	-0.027
Voluntary activities 2015	0.081	-0.001	0.119	0.016	0.103	0.062***	0.101	0.027
Voted in elections 2014	0.790	-0.011	0.829	0.035	0.716	0.014	0.783	0.005

The table presents results of matching estimation for women stratified by number of years in the education system and past SA. For each stratum and each outcome variable, two values are reported: value of the outcome variable for the treated group (i.e. active in 2013; left column) and estimated difference between the treated group and the non-treated group (i.e. inactive in 2013; right column). For the latter, significance is denoted using asterisks

*** p < 0.010

** p < 0.050

* p < 0.100. For the sample sizes, only observations on common support are reported (3.9–10% observations were off common support–with the highest for the active low-education group).

Nevertheless, for women with more than 12 years in education, a positive effect of initiating SA in 2013 on general trust can be observed—the ratio of women declaring that most people can be trusted increases by 0.116 to 0.270 (p < 0.001).

For men, the positive impact of SA on health can be observed. Initiating SA in 2013 meant greater satisfaction with health–both for men over 40 (3.053 vs 3.221 against a reverted, 1–6 scale, p = 0.060) and those under 40 (2.326 vs 2.656, p < 0.001). For the older age group, sustaining SA in 2013 had a comparable effect (2.95 vs 3.233, p = 0.012). Further effects for men under 40 can be observed in the case of sustaining activity in 2013 as a lower ratio of men declaring physical problems (0.347 vs 0.499, p = 0.008) and becoming seriously ill in the year before the SD survey (2014) less often (0.050 vs 0.102, p = 0.016). Subsequently, for men over 40, initiating SA in 2013 meant greater chances to admit to overusing alcohol in the year before the SD survey (0.183 vs 0.117, p = 0.005), but the opposite effect can be seen for men sustaining SA in 2013 (0.071 vs 0.127, p = 0.031).

Furthermore, similar to women (Table 1), a positive impact on life attitudes can be observed in men (Table 3).

**Table 3:** Effects of sports activity (‘treatment’) on men belonging to different age groups.

Outcome variable	Age 41–64 non-active men (n = 1,376)		Age 41–64 active men (n = 539)		Age 25–40 non-active men (n = 547)		Age 25–40 active men (n = 367)	
	treatment group (n = 248)	difference	treatment group (n = 347)	difference	treatment group (n = 153)	difference	treatment group (n = 244)	difference
*Sport participation*								
Sport 2015	0.305	0.197***	0.703	0.382***	0.392	0.182***	0.751	0.442***
>1 category of sport activity 2015	0.073	0.051**	0.272	0.155***	0.160	0.117***	0.394	0.246***
*Health and physical conditions*								
BMI 2015	27.926	-0.152	27.915	-0.314	26.696	-0.356	26.799	-0.189
Dissatisfaction with health 2015	3.053	-0.168*	2.946	-0.287**	2.326	-0.330***	2.397	-0.055
Physical problems 2015	0.758	-0.009	0.715	-0.067	0.486	-0.006	0.347	-0.152***
Health problems 2015	0.693	0.026	0.651	-0.035	0.382	-0.022	0.357	0.047
Seriously ill 2014	0.140	-0.013	0.170	0.049	0.031	-0.034*	0.050	-0.052**
Tiredness not-related to work 2015	0.428	0.025	0.442	0.052	0.325	0.010	0.276	0.050
Too much alcohol 2014	0.183	0.066***	0.071	-0.056**	0.089	-0.038	0.097	-0.021
*Life attitiudes*								
No decrease in energy to work 2015	0.612	.085**	0.670	0.036	0.823	0.019	0.860	0.079*
Entire life delightful or pleasing 2015	0.438	0.043	0.515	0.039	0.454	0.003	0.582	0.111*
Lust for life 2015	8.533	0.147	8.624	-0.217	8.876	0.289*	9.129	0.478***
Achieving goals > fun 2015	0.533	-0.043	0.626	-0.039	0.524	0.014	0.533	0.008
Success depended on her/himself 2015	0.801	0.100***	0.807	0.088**	0.846	0.061	0.842	0.034
Fun is the most important thing 2015	0.654	0.025	0.598	-0.029	0.733	0.073*	0.671	-0.162***
*Social activities*								
Member of sports club 2015	0.004	0.001	0.044	0.031**	0.002	-0.004	0.060	0.057***
Number of friends 2015	7.328	1.586***	8.518	-0.116	5.928	0.487	6.918	1.628*
Number of acquaint. met regularly 2015	5.807	0.421	7.909	-0.976	7.683	0.984	8.598	1.329
Number of friends met regularly 2015	4.744	0.371	5.962	0.057	5.385	0.652	6.105	0.870*
Most people can be trusted 2015	0.151	0.020	0.137	-0.054*	0.173	0.048	0.242	0.067
Work for local society 2013–2014	0.204	0.059**	0.334	0.048	0.190	0.071**	0.216	0.039
Member of organisations 2015	0.154	0.034	0.433	0.137**	0.090	-0.021	0.245	0.157***
Took part in public meeting 2015	0.231	0.002	0.345	0.015	0.196	0.007	0.228	0.050
Voluntary activities 2015	0.113	0.059***	0.124	-0.050	0.083	0.026	0.092	0.010
Voted in elections 2014	0.722	-0.029	0.839	0.005	0.715	0.080*	0.700	-0.023

The table presents results of matching estimation for men stratified by age and past SA. For each stratum and each outcome variable, two values are reported: value of the outcome variable for the treated group (i.e. active in 2013; left column) and estimated difference between the treated group and the non-treated group (i.e. inactive in 2013; right column). For the latter, significance is denoted using asterisks

*** p < 0.010

** p < 0.050

* p < 0.100. For the sample sizes, only observations on common support are reported (5.6–13.4% observations were off common support–with the highest ratio for active under-40).

Notably, men over 40 tend to attribute their success to their own efforts, after either sustaining (0.807 vs 0.719, p = 0.030) or initiating SA in 2013 (0.801 vs 0.701, and p < 0.001). For men over 40 initiating SA in 2013, the probability of declaring no decrease in energy to work is higher (0.612 vs. 0.527; p = 0.017). For men under 40, two effects of sustaining SA in 2013 can be seen: greater lust for life (9.129 vs 8.651 against a 1–10 scale, p = 0.009) and a lower ratio of respondents declaring that having a lot of fun is the most important thing in life (0.671 vs 0.833, p < 0.001).

In terms of social activity, the effects for men are less clear than for women. Nevertheless, men over 40 initiating SA in 2013 also declared having more friends (on average, 7.328 vs 5.742, p = 0.003), they more often worked for the benefit of local society (0.204 vs 0.145, p = 0.042) and engaged in voluntary activities (0.113 vs 0.054, p = 0.002). For men of all ages, a positive impact of sustained SA on the probability of being an active member of a sports club can be seen (0.044 vs 0.013 for older men, p = 0.024; 0.060 vs 0.003 for younger men, p = 0.001).

Like those for women, the results for men based on stratification using number of years in education (Table 4) were mostly consistent with those presented above.

**Table 4:** Effects of sports activity (‘treatment’) on men with different number of years in education.

Outcome variable	High edu level non-active men (n = 444)		High edu level active men (n = 446)		Low edu level non-active men (n = 1,449)		Low edu level active men (n = 379)	
	treatment group (n = 127)	difference	treatment group (n = 314)	difference	treatment group (n = 263)	difference	treatment group (n = 192)	difference
*Sport participation*								
Sport 2015	0.469	0.235***	0.812	0.417***	0.294	0.192***	0.634	0.424***
>1 category of sport activity 2015	0.261	0.186***	0.402	0.24***	0.066	0.055***	0.177	0.134***
*Health and physical conditions*								
BMI 2015	27.069	-0.265	27.204	-0.430	27.730	-0.158	27.909	0.008
Dissatisfaction with health 2015	2.608	-0.145	2.758	-0.038	2.996	-0.160*	2.704	-0.288**
Physical problems 2015	0.630	0.054	0.507	-0.089*	0.658	-0.060*	0.637	-0.056
Health problems 2015	0.548	0.067	0.504	-0.008	0.604	-0.025	0.596	0.023
Seriously ill 2014	0.089	-0.006	0.101	0.048**	0.117	-0.023	0.150	0.012
Tiredness not-related to work 2015	0.480	0.174***	0.328	-0.012	0.392	-0.002	0.431	0.061
Too much alcohol 2014	0.075	-0.035	0.097	0.006	0.140	0.018	0.086	-0.019
*Life attitiudes*								
No decrease in energy to work 2015	0.744	-0.014	0.820	0.078*	0.652	0.097***	0.691	0.051
Entire life delightful or pleasing 2015	0.559	0.060	0.578	0.057	0.424	0.054	0.486	0.085
Lust for life 2015	8.847	0.026	8.961	0.139	8.573	0.282**	8.753	0.180
Achieving goals > fun 2015	0.565	0.012	0.623	-0.005	0.563	0.010	0.544	-0.023
Success depended on her/himself 2015	0.910	0.080**	0.877	0.018	0.812	0.114***	0.781	0.091*
Fun is the most important thing 2015	0.555	-0.032	0.613	-0.057	0.745	0.083***	0.654	-0.084*
*Social activities*								
Member of sports club 2015	0.004	-0.011	0.069	0.030	0.005	0.004	0.015	0.015
Number of friends 2015	6.615	0.674	7.017	0.860	7.172	1.564***	8.777	2.769***
Number of acquaint. met regularly 2015	8.647	2.562**	8.404	0.380	6.064	0.555	7.701	2.733***
Number of friends met regularly 2015	5.413	0.388	5.977	0.297	5.082	0.692**	5.809	1.352***
Most people can be trusted 2015	0.148	0.030	0.211	-0.016	0.162	0.026	0.157	0.038
Work for local society 2013–2014	0.331	0.154***	0.299	-0.041	0.194	0.080***	0.257	0.125***
Member of organisations 2015	0.224	0.008	0.412	0.070	0.089	0.009	0.282	0.157***
Took part in public meeting 2015	0.262	-0.008	0.300	-0.078	0.192	-0.004	0.302	0.144***
Voluntary activities 2015	0.119	0.059**	0.114	-0.047	0.069	0.016	0.099	0.041
Voted in elections 2014	0.772	0.031	0.813	0.016	0.669	-0.031	0.772	0.047

The table presents results of matching estimation for men stratified by number of years in education system and past SA. For each stratum and each outcome variable, two values are reported: value of the outcome variable for the treated group (i.e. active in 2013; left column) and estimated difference between the treated group and the non-treated group (i.e. inactive in 2013; right column). For the latter, significance is denoted using asterisks

*** p < 0.010

** p < 0.050

* p < 0.100. For the sample sizes, only observations on common support are reported (3.7–18.3% observations were off common support–with the highest for the active low-education group).

The positive effect of SA on the relational and organisational dimension of social capital is more evident for the stratification based on education (Table 4), not age (Table 3). For example, men with a low level of education sustaining SA in 2013 had more friends than men ceasing SA (on average, 8.777 vs. 6.008, p < 0.001), met more friends (on average, 7.701 vs. 4.968, p = 0.008) and acquaintances (on average, 5.809 vs. 4.457, p = 0.002), were members of more organisations (on average, 0.282 vs. 0.125, p = 0.005), and more of them worked for the benefit of local society (0.257 vs. 0.132, p = 0.003) and took part in public meetings (0.302 vs. 0.158, p = 0.001).

## Discussion

Strengthening the health potential of society requires recognition of various factors and mechanisms responsible for human behaviour, which—through the synergy effect—can support this process. Considering that sports activity (SA) can affect human health (physical, mental, social and spiritual) [39], it is necessary to recognize the causal relationship of this activity, not only through already well-documented health/physical conditions [17,20], but also through various aspects of the social environment in people’s lives (such as social cohesion or social capital) [7], and with their attitudes to life [10]. However, it is important to consider the sustainability of involvement in SA. Understanding the powerful interaction of a combination of these factors is one of the ways to consciously explain the health outcomes.

The presented results indicate that the self-reported health and physical conditions, life attitudes and social activity of the respondents mostly depend on whether and how they were involved in SA (whether it was an activity just initiated or systematically undertaken for a longer time).

The first, most consistent and robust outcome is the persistence of SA. For most of the strata, respondents active in 2013 were twice or three times as likely to remain so in 2015 (in comparison with non-active ones in 2013)–these results find scientific confirmation in other studies [40]. This is supported by the worldwide increase of sports participation (maintained since the 1970s [41, 42, 43], and growing over the last decade) [39]. In our study, this strong tendency is confirmed by the increasing probability of engaging in more than one form of SA, as it is sustained (both among high and low educated women and men). This indicates that in all examined groups, SA is a self-reinforcing process and neither age nor level of education significantly affects this rule, although, according to previous reports [43], the probability of SA is higher in better educated people. Scheerder and Vos [43] pointed out a similar phenomenon. They showed an increase in sports participation, at all levels of education, in recent years, explaining this fact with an increase in the level of education worldwide—which is associated with better knowledge and awareness of the need to initiate physical activity.

In our sample, a substantial heterogeneity was observed in respect of how participation in sports influences people’s lives. As we can see, SA affects self-reported health and physical condition. However, this impact depends on whether the SA was recently initiated (in 2013) or was sustained (started in 2011 and continued until 2013). Boyette et al. [44] believe that older adults who are in good health and have a history of exercise might be more likely to participate in long-term exercise programmes. Nevertheless, this does not indicate the causality of this phenomenon.

Our results prove that SA initiation results in a lower dissatisfaction with health (in relation to those who were not active in 2011 and remained inactive in 2013). This applies to all groups, both > 40-year-olds (men—p = 0.060 and women—p = 0.021) as well as < 40-year-olds (men—p < 0.001 and women—p < 0.001). This might result from the fact that the initiation of physical exercise evokes a general improvement in well-being [45] and a positive perception of health and body [46], i.e. general energy increase or, after exercising, resolution of everyday complaints such as back pain, shortness of breath, or muscle tension.

Research shows that even 15 minutes of moderate-to-vigorous exercise can have important benefits (for example, for both affective experience and cognitive performance, regardless of age [47]). Similar effects were noted in the reported physical problems, although this was true for younger women (< 40 years) only. However, women initiating SA in 2013 declared such problems less frequently (p = 0.014) than their inactive peers. Regardless of age, all women also declared tiredness not related to work less often (p = 0.008). This was particularly visible among those with a high level of education (over 12 years of education, p < 0.001). It is not easy to explain this fact. One of the assumptions is that (even though they were asked about tiredness not related to work) it is a consequence of their labour (mental, non-physical). However, fatigue is the state of weariness after a period of exertion, mental or physical, characterized by a decreased capacity for work and reduced efficiency in responding to stimuli [48]. Thus, it applies equally to people with high and low levels of education and can equally affect their well-being and health [49]. Perhaps, this is the result—emphasized by many scientists [50]- of more frequent participation in sport of people with higher education and the fact that regular physical activity can help reduce fatigue for many people [51].

SA initiation among men (especially those aged > 40 years) meant more frequent overuse of alcohol (p = 0.005), but the opposite effect was observed among those sustaining SA (p = 0.031). The fact that physical activity and alcohol consumption are interrelated is quite well known [52]. Many studies indicate higher rates of alcohol intake among athletes—in comparison with non-athletics peers [53]. A common explanation is that athletes celebrate their victories (or failures) together, and gathering in associations, clubs and teams encourages this type of behaviour [54]. As a result, among people who exercise, not necessarily in sports clubs, indicators of risky alcohol consumption are higher than in the general population [55]. Lisha et al. [52] indicate that this relationship is the strongest in men (especially up to 50-year-olds, who train intensively). Our results do not confirm this trend and prove that sustaining SA reduces the likelihood of overusing alcohol. Long-term SA is probably associated with an increase in knowledge about pro-health behaviour and a greater ability to assess these behaviours. It is easier to see the problem of overusing alcohol when you exercise and need to take care of your physical condition and the willingness to change behaviour becomes stronger.

The health effects of sustaining SA (in 2011 and in 2013) for declared health and physical condition were visible only in the case of men. This suggests that the pro-health impact of systematic SA is more pronounced in men than in women, and that perhaps (which we do not see in the analyses) women see other values ​​in systematic SA, like general activation or lifestyle, which are important for them. This assumption is confirmed by Borgers et al. [42] and Allender et al. [56], who state that women are more likely than men to achieve pleasure and social interaction. However, inactive men aged > 40 years better assess dissatisfaction with health (p = 0.012), and men aged < 40 years are less likely to report physical problems (p = 0.008) and serious illness (p = 0.016) than inactive ones. In light of the evidence regarding the health benefits of systematic physical activity, these results are quite obvious. Eberth and Smith [57] speak directly about creating one’s own health, and Leasure et al. [54] about the fact that protective factors tend to accumulate.

People who engage in one pro-health behaviour, such as practising sport, are also involved in other health behaviours (e.g. limiting alcohol consumption). More often they pay attention to proper nutrition, regular medical check-ups, stimulant avoidance, regular rest and sleep hygiene [58]. However, these behaviours are very well established and fundamental. This is, among other things, the result of expectations, predictions, beliefs, individual thinking, and emotional mechanisms of the personality [59], which is confirmed by psychological studies describing the importance of life attitudes in each phase of the process of changing health behaviours [60].

The analysis of this phenomenon requires a double-sided perspective, mentioned by authors cited above, that the pro-health behaviours result from individual and social factors, and, from another perspective mentioned by other authors [61, 62], that social relations can be an important component of health. It is proven that factors such as social status, a sense of control over one's life and work, social support network, education, self-esteem, and civic activity cause real differences in the health of the community, and that completeness and regularity of social relations are important for health [61]. Thus, the results of our research indicate that SA initiation affects respondents’ attitudes. It was found that the probability of declaring a decrease in energy for work is lower among women who initiated SA in 2013 (< 40 years—p = 0.053 and > 40 years—p < 0.001), compared to their inactive peers. This is visible both in the case of high and low educated women (p = 0.021 and p < 0.001, respectively). They are also more likely to be optimistic about the future, especially women over 40 years of age (p = 0.003) and low educated (p = 0.003). Among women aged < 40 years, the initiation of activity results in a strong increase in life satisfaction (p = 0.001).

VanDam [63] argues that positive life attitudes cannot stop a serious illness. Similarly, Friedman et al. [64] have doubts about the protective effect of happiness, pointing to higher death rates among happy people due to their lifestyle, which is riskier than others. However, according to Veenhoven [65], happy people make better choices in life, because they are more open to the world and more self-confident. People who regularly practise sports are characterized by a stronger sense of happiness and optimism [65]. Researchers show that optimism has a very positive effect on human physical and mental health and motivates people to undertake pro-health behaviours [66]. Similar relationships might be observed concerning self-efficacy. Together with an increase in the belief in the possibility of achieving goals is the motivation to increase a specific behaviour [66]. In socio-cognitive theory, self-efficacy has a clear impact on people's behaviour, determining the choice of goals (the stronger the effectiveness, the more ambitious goals) and conditioning the expected benefits and losses. Stronger self-efficacy fosters the perception of benefits [67]. Apart from this, the high self-esteem of one’s own effectiveness favours more rapid processing of information, better task execution, formulating goals and achieving successes, whereas low self-efficacy combines with anxiety, helplessness and the intensity of sadness and depression. It can, therefore, affect self-esteem and, as a result, mental health [67]. Proven by Christiansen et al. [68], the positive relationship between SA and self-esteem suggests further research in this domain is necessary. Our results show that the initiation of SA in men affects a more frequent belief that success depends on oneself, especially those aged > 40 years (p < 0.001) and with a low level of education (p < 0.001).

The effect of the continuation of SA is a higher probability of declaring a decrease in energy to work among older respondents (> 40 years), for both women (p = 0.015) and men (p = 0.017). In the group of older men, there are more frequent declarations that success depends on oneself (p = 0.030), and in younger ones, two effects of maintaining SA were a greater lust for life (p = 0.009) and a lower percentage declaring that having a lot of fun is the most important thing in life (p <0.001).

Analysing personality factors that are important in health behaviours, researchers often focus on the determinants of adverse health behaviours, referred to as anti-health or even risk (i.e. smoking, alcohol or drug use). The personality traits that support risk behaviours are emotional immaturity, low self-esteem and low resistance to stress, excessive dependence, difficulties in expressing feelings, a high level of anxiety and perceived isolation combined with a lack of trust [69]. Lack of trust reduces the quality of mental health and fosters depression, to which people with less emotional support are more vulnerable. Women in this group represent a higher risk of pregnancy complications and are more likely than others to suffer disability after having a chronic illness [62]. Exclusion from formal and informal social networks is bad for overall health, and reduced SC is associated with a higher risk of myocardial infarction [16]. The presented results suggest that SA can positively influence the generation of social capital, reinforcing the results obtained by Schüttoff et al. [25] for adolescents. Like their outcome, as well as these of Kim et al. [22] or Legh-Jones and Moore [24], it is the social network aspect–in our case, number of friends, friends or acquaintances met–that is most profoundly related to sports participation. This applies especially to women aged > 40 years who initiated AS in 2013. They declared a greater number of friends (p = 0.001, especially those who are low educated p = 0.014) and number of friends met regularly (p = 0.003).

In the opinion of scientists, building satisfying interpersonal relations, good communication skills, making social contacts and resolving conflicts serve to maintain social health [67]. At the same time, however, network, inter-group solidarity and shared resources support the enforcement and strengthening of social health-promoting norms and provide tangible, substantive support [7, 70]. Therefore, people with a higher sense of coherence lead a healthier lifestyle [71]. Our research also shows a positive impact on other dimensions of social activity. For example, more women > 40 years of age initiating SA in 2013 work for the local community (p < 0.001), participate in public meetings outside of work (p = 0.015) and engage in voluntary activities (p < 0.001), and more women (on average) are members of more organizations (p = 0.005). Each of these cases often applies to low educated women (p = 0.001, p = 0.021, p = 0.001, p = 0.007, respectively).

Women with over 12 years of education more often perceive that people can be trusted (p < 0.001). Ball et al. [23] confirm that physical activity (requiring women to leave the house) is linked with reciprocal trust (at the neighbourhood level) and with social activity. Lindstroem et al. [72] also indicate a similar relationship. According to Son et al. [73], physical activity fosters the building of such SC elements as bonding and bridging opportunities, social support, community engagement and volunteering. In the authors’ opinion, these elements combine with individual and social health and well-being.

Although the literature indicates that women are generally more social and more oriented towards maintaining social relations than men [74], in our study the social effects of SA initiation in 2013 are generally less pronounced for men than for women. Men initiating SA (especially those aged > 40) tend to have more friends than those who are inactive (p = 0.003), more often work for the local community (p = 0.042) and engage in voluntary activities (p = 0.002). In all men, however, the positive effect of sustaining SA on the probability of being an active member of a sports club is clear (among older—p = 0.024 and younger—p = 0.001). Eime et al. [39] confirm that a significant part of organized participation in a sports club environment is more likely for men than for women.

A study on children in Germany showed that there was a positive and statistically significant relationship between belonging to a sports club and social acceptance and positive perception by peers [75]. Perhaps this is the reason why some of our male respondents participate in organized sport. Women are more interested in unorganized sport, which is related to a new lifestyle and new social trends [76].

Sport in the modern world has transformed into an industry of its own [76]. This is connected with the transition from understanding sport as "exceptional" to normative behaviour; with the abolition of the image of the athlete attributed only to young people or men; with the reduction of social structures related to sport; with the expansion of services and possibilities of participating in alternative and recreational forms as well as the diversity of SA motives [42].

Women might be more interested in meeting friends and in teamwork for achieving goals than a sports score (or previously mentioned health improvement). This may be indirectly indicated by the fact that women (aged > 40) who sustain SA more often declare a greater number of friends (p = 0.011) and acquaintances met regularly (p = 0.040). Studies show that being with friends makes it easier to initiate sport [77]. In addition, this does not require long-term involvement (as is the case with traditional club athletes), but gives the chance to participate in unstructured, short-term activities [43], which is quite important for women [56, 42]. Perhaps in women this means frequent “switching” of physical activity types/forms.

The positive impact of SA on the relational and organizational dimension of SC is visible more from the perspective of the respondents’ education than in their age. The analysis shows that low educated men sustaining AS in 2013 have more friends than men who have stopped being active (p = 0.008). They also declare a larger number of friends met regularly (p = 0.002), they are members of a larger number of organizations (p = 0.005), more of them work for the benefit of local communities (p = 0.003) and participate in public meetings (p = 0.001).

Eberth and Smith [57] showed that communities with a large SC are more aware of the positive effects of SA, and therefore are more eager to initiate it. Kramer et al. [50] add that such communities can strengthen positive social norms for health behaviours, through the effect of “infection”, for instance [78].

## Conclusions

The results of our research indicate that sports activity can be a tool for the policy of social activation and strengthening health potential.

In line with expectations, our analysis has shown that engagement in SA has a strong self-reinforcement effect. Not only does initiating it increase the probability of continuing, but ceasing it reduces chances to continue further–this effect is true for all strata examined. The pseudo-experimental design striving to establish causal relations matters in this respect. This result suggests that even random (conditional on controlled characteristics) initiating or ceasing SA (but long enough to lead the respondents to declare such events in 2013 –possibly, lasting a few months) often also has an impact on SA two years later. Therefore, not only certain long-term personal characteristics can influence propensity to engage in sport, but SA also propagates itself.

In terms of self-reported health, social activity and attitude, the effects of initiating SA are usually stronger than of sustaining/ceasing it. Social involvement is raised more profoundly in the case of women than men and in the case of individuals over 40.

A relatively consistent result is that SA leads to increasing and strengthening social relations, so that not only the number of people regularly met increases, but also the number of perceived friends. Therefore, SA is shown as a tool with the potential to enhance social health, especially for people over 40.

### Limitations

Finally, we should emphasise the limitations of the data utilised. In particular, Lechner and Sari [36] show that positive effects are mostly associated with ‘intensive’ (i.e. with daily energy expenditure above 3 kcal/kg) participation in sports, while Lechner [35] and Schüttoff et al. [25] show results for ‘regular’ participation (in the first case, at least monthly, in the other, at least weekly). Unfortunately, SD design, quite understandably, does not include such information. Combining any other sports-specific dataset with SD is impossible due to anonymised samples (and, most likely, consisting of different participants).

Furthermore, due to SD, unfortunately, being discontinued after 2015, it is impossible to investigate the impact of SA in the longer term. This is particularly important when one considers that some of the positive effects of SA reported in the literature (on labour market outcomes) mostly take place after more than five years. Nevertheless, our results (in terms of social involvement, including relations with other people) might indicate one of the important channels of such an impact.

## Supporting information

S1 Appendix Coding of variables. (DOCX)

S2 Appendix Descriptive statistics. (DOCX)

S3 Appendix Results of the probit model estimations. (DOCX)

S4 Appendix Selective panel attrition. (DOCX)

S5 Appendix Additional stratifications. (DOCX)

S6 Appendix Estimation results for key outcomes—Effects of different technical assumptions. (DOCX)

S1 File Final database. (ZIP)

S2 File Labels for the final database. (CSV)
