# Supplementary material for: Impact of sports activity on Polish adults: Self-reported health, social capital & attitudes
Source: PLoS One. 2019 Dec 19;14(12):e0226812. doi: 10.1371/journal.pone.0226812 (PMC6922371; doi:10.1371/journal.pone.0226812)
Supplement: S1 Appendix — (DOCX) [file pone.0226812.s001.docx]

# S1 Appendix. Coding of variables.

**Table A.** **Variables based on the Social Diagnosis.**

| **Variable** | **Question** | **Possible answers** | |
| --- | --- | --- | --- |
| Treatment variable: Sport activity | Do you practice any sport or physical activity? | 1. no, I do not practice any sport or physical activity  2. aerobics  3. running/jogging/nordic walking  4. gym  5. cycling  6. skiing or other winter sports  7. swimming  8. football or other team sports  9. yoga  10. martial arts  11. another sport or type of physical activity.  Recoded as binary variable  (2-11 as 1) | |
| >1 category of sport activity | As above | Recoded as binary variable: 1 when at least two out of 2-11 chosen | |
| Too much alcohol | In the last year, have you drunk too much alcohol | Yes; No | |
| Drugs/designer drugs | In the last year, have you tried drugs/designer drugs | Yes; No | |
| Number of cigarettes | Do you smoke?  if YES, how many cigarettes a day do you smoke on average? | Yes; No + Open-ended question (integer) (No recoded as 0) | |
| Friends met regularly | How many persons you contact with regularly for social and personal reasons (at least several times a year), among friends? | Open-ended question (integer) | |
| Acquaintances met regularly | How many persons you contact with regularly for social and personal reasons (at least several times a year), among acquaintances (work/school colleagues, neighbors and others)? | Open-ended question (integer) | |
| Family members met regularly | How many persons you contact with regularly for social and personal reasons (at least several times a year), among close family members? | Open-ended question (integer) | |
| Friends | How many persons do you consider to be your friends? | Open-ended question (integer) | |
| Work for local society | During the last two years, have you been involved in any actions for the benefit of your local  community (gmina, housing estate, town or neighborhood)? | Yes; No | |
| Member of organisations | Are you a member of any organizations, associations, parties, committees, councils, religious groups or clubs? | 1. Yes, one; 2. Yes, two; 3. Yes, three or more; 4. No  (4 recoded as 0, 1 as 1, 2 as 2, 3 as 3) | |
| Fullfiling roles in organisations | Are you a member of any organisations, associations, parties, committees, councils, religious groups or clubs?  — if YES, have you hold any roles in such organisations? | 1. Yes, one; 2. Yes, two; 3. Yes, three or more; 4. No  + binary (recoded 4 for the first question as 0) | |
| Member of a sports club | Do you currently take active part in sports club? | Yes; No | |
| Took part in public meeting | Have you attended any public meeting in the last year (but not at your workplace)? | Yes; No | |
| Voluntary activities | Have you in the last year done any voluntary work for people outside the family or for a social  organization? | 1. Yes, often; 2. Yes, rarely; 3. No  (recoded as a binary variable, 1 and 2 as 1; for 2011 only 1: Yes and 2: No - 1 as 1) | |
| Voted in 2010 | Did you vote in the last self-government elections in 2010? | 1. Yes; 2. No;  (recoded as a binary variable, 1 as 1) | |
| Voted in 2014 | Did you vote in the last self-government elections in 2014? | 1. Yes; 2. No; 3. I was under 18 years of age  (recoded as a binary variable, 1 as 1) | |
| Number of social meetings | In the last month, how many times have you gone to meeting with friends | Open-ended question (integer) | |
| Number of entertainment | In the last month, how many times have you gone to cinema, theatre, concert | Open-ended question (integer) | |
| Number of restaurants | In the last month, how many times have you gone to restaurant, cafe, pub | Open-ended question (integer) | |
| Afraid of neighbourhood | In the recent months, you have feared because of crime, drug addiction and hooliganism in your district, housing estate or neighbourhood. | 1. Often  2. Once or twice  3. Never  Recoded as a binary variable (1 as 1) | |
| Lust for life | At present, how strong is your willingness to live? | 1: I do not want to live at all, 2, 3,..., 10: I want to live very much | |
| Achieving goals > fun | What, in your opinion, is most important in life? | 1. fun, well-being, lack of stress; 2. sense of purpose, achieving important goals despite difficulties, pain and sacrifice  Recoded as a binary variable (2 as 1) | |
| Success depended on her/himself | Who or what had impact on the last year being a good one or a bad one for you? (more than one answer may  be checked) | 1. authorities  2. myself  3. other people  4. fate (providence)  Recoded as a binary variable (Yes to 2 as 1) | |
| Belief in democracy | With which of the following statements on democracy do you agree most? | 1 democracy is a superior form of governance  2 sometimes non-democratic rule is better than democracy  3 it does not really matter whether the government is democratic or not  4 democracy is a bad form of government  5 it is hard to say  Recoded as a binary variable (1 as 1) | |
| Supports Law and Justice | Which of the political parties do you sympathise with most? (in the brackets – the name of the party leader) | 1 Law and Justice, PiS (Jarosław Kaczyński)  2 Polish People‘s Party, PSL (Waldemar Pawlak)  3 Democratic Left Alliance, SLD (Grzegorz Napieralski)  4 Poland Comes First, PJN (Joanna Kluzik-Rostkowska)  5 Civic Platform, PO (Donald Tusk)  6 other  7 none  8 it is hard to say  Recoded as a binary variable (1 as 1) | |
| Supports no political party | Which of the political parties do you sympathise with most? (in the brackets – the name of the party leader) | 1 Law and Justice, PiS (Jarosław Kaczyński)  2 Polish People‘s Party, PSL (Waldemar Pawlak)  3 Democratic Left Alliance, SLD (Grzegorz Napieralski)  4 Poland Comes First, PJN (Joanna Kluzik-Rostkowska)  5 Civic Platform, PO (Donald Tusk)  6 other  7 none  8 it is hard to say  Recoded as a binary variable (7 as 1) | |
| Fun is the most important thing | *Below you will find several statements. Please specify to what extent these statements match your beliefs and attitudes. Provide your opinions by entering the selected digit in the appropriate box*  In life the most important thing is to have a lot of fun. | 1. Definitely yes; 2. Yes; 3. Rather yes; 4. Neither yes nor not; 5. Rather not; 6. No; 7. Definitely not  Recoded as a binary variable (1, 2 and 3 as 1) | |
| Entire life delightful or pleasing | How do you perceive your entire life? Could you say it was… (please cross the appropriate box) | 1 delighted  2 pleased  3 mostly satisfying  4 mixed  5 mostly dissatisfying  6 unhappy  7 terrible  Recoded as a binary variable (1 and 2 as 1) | |
| Money - 1 of 3 most important things in life | What in your opinion is the most important condition of a successful, happy life (please first read through all the answers and then choose no more than three, by crossing the appropriate boxes): | 1 money  2 children  3 successful marriage  4 work  5 friends  6 providence, god  7 cheerfulness, optimism  8 honesty  9 kindness and being respected  10 freedom  11 good health  12 education  13 strong personality  14 other  Recoded as a binary variable (Yes yo 1 as 1). | |
| Reforms in Poland successful | In your opinion, were the reforms in Poland after 1989 in general successful or unsuccessful? | 1 successful  2 unsuccessful  3 it is hard to say Recoded as a binary variable (1 as 1) | |
| Losing interest in sex | Read the four statements in each point carefully and then choose one  that describes best your feelings and beliefs during the last month | 0. I have not noticed any recent change in my interest in sex; 1. I am less interested in sex than I used to be.; 2. I am much less interested in sex now.; 3. I have lost interest in sex completely. Recoded as a binary variable (1-3 recoded as 1). | |
| Dissatisfaction with sex life | To what extent are you satisfied with your sex life? | 1: very satisfied, 2: satisfied, ... 6: very not satisfied, 7: not applicable | |
| BMI | Calculated based on answers to open-ended questions: How tall are you? (cm); What is your weight? (kg) | | |
| Dissatisfaction with health | To what extent are you satisfied with your health condition? | 1: very satisfied; 2: satisfied; ... 6: very not satisfied, 7: not applicable | |
| Problems with sleeping | Read the four statements in each point carefully and then choose one that describes best your  feelings and beliefs during the last month. | 0. I sleep at least as well as I used to.  1. I do not sleep as well as I used to.  2. In the morning, I wake up 1-2 hours earlier and find it difficult to fall asleep again.  3. I wake up several hours too early and I can‘t get back to sleep.  Recoded as a binary variable (2 and 3 as 1). | |
| Some worries about digestive system | Read the four statements in each point carefully and then choose one that describes best your  feelings and beliefs during the last month. | 0. I am not worried about my health any more than I used to be.  1. I am worried about such ailments as: stomach pains, upset stomach, or constipation.  2. I am very worried about my health; I think about it constantly.  3. My health condition is so worrying that I cannot think of anything else.  Recoded as a binary variable (1 as 1). | |
| Constant worries about health | Read the four statements in each point carefully and then choose one that describes best your  feelings and beliefs during the last month. | 0. I am not worried about my health any more than I used to be.  1. I am worried about such ailments as: stomach pains, upset stomach, or constipation.  2. I am very worried about my health; I think about it constantly.  3. My health condition is so worrying that I cannot think of anything else.  Recoded as a binary variable (2 and 3 as 1). | |
| Health problems | In the recent months your health problems have made it difficult for you to perform everyday activities or to take part in other activities? | 1. Often  2. Once or twice  3. Never  Recoded as a binary variable (1 and 2 as 1) | |
| Physical problems | In the recent months you have suffered from ailments, such as bones aching or shortness of breath, etc., which has made it difficult for you to leave home, climb the stairs, etc? | 1. Often  2. Once or twice  3. Never  Recoded as a binary variable (1 as 1) | |
| Seriously ill | In the last year have you been seriously ill? | Yes; No | |
| Headaches | Below you will find a list of some ailments. Please specify whether you suffered from them LAST MONTH: strong headaches | 1. I did not suffer.  2. I suffered for less than 15 fays  3. I suffered at least for half a month  Recoded as a two binary variables: Headaches (<1/2) (2 as 1)  Headaches (>1/2) (3 as 1) | |
| Stomach pains | (as above): stomach pains or flatulence | as above | |
| Pain in neck or arm muscles | (as above): pain or tension in the neck or arm muscles | as above | |
| Chest or heart pains | (as above): chest or heart pains | as above | |
| Dry mouth or throat | (as above): dry mouth or throat | as above | |
| Sweating | (as above): attacks of excessive sweating | as above | |
| Shortness of breath | (as above): shortness of breath | as above | |
| Body pains | (as above): pains throughout the whole body | as above | |
| Palpitation | (as above): accelerated heartbeat (palpitation) | as above | |
| Shivers or convulsions | (as above): shivers or convulsions | as above | |
| Pressure on bladder | (as above): pressure on the bladder and more frequent urinating | as above | |
| Tiredness | (as above): a feeling tiredness not associated with work | as above | |
| Constipation | (as above): constipation | as above | |
| Nosebleeds | (as above): nosebleeds | as above | |
| Blood pressure | (as above): sudden changes of blood pressure | as above | |
| Rare suicidal thoughts | In the recent months, how often have you been so depressed you have thought about suicide? | 1. Very often; 2. Rather often; 3. Rarely; 4. Never  Recoded as a binary variable (3 as 1) | |
| Often suicidal thoughts | In the recent months, how often have you been so depressed you have thought about suicide? | 1. Very often; 2. Rather often; 3. Rarely; 4. Never  Recoded as a binary variable (1 and 2 as 1) | |
| Age | Pre-coded in the database – based on date of birth | | |
| Years of education completed | Pre-coded in the database – based on direct question | | |
| Higher Education | Based on information on educational attainment pre-coded in the database (1 if higher education with at least a PhD title, higher education with at least an MA degree or an equivalent degree, higher education with an Engineer or Bachelor degree) | | |
| English | Command of foreign languages: English | | 1. active; 2. passive; 3. none.  Receded as a binary variable (1 as 1) |
| German | Command of foreign languages: German | | 1. active; 2. passive; 3. none.  Receded as a binary variable (1 as 1) |
| French | Command of foreign languages: French | | 1. active; 2. passive; 3. none.  Receded as a binary variable (1 as 1) |
| Russian | Command of foreign languages: Russian | | 1. active; 2. passive; 3. none.  Receded as a binary variable (1 as 1) |
| Spanish | Command of foreign languages: Spanish | | 1. active; 2. passive; 3. none.  Receded as a binary variable (1 as 1) |
| other language | Command of foreign languages: other language | | 1. active; 2. passive; 3. none.  Receded as a binary variable (1 as 1) |
| Educated her/himself | During the last 2 years, has this person participated in any activity related with gaining new professional qualifications or other skills? | | Yes; No |
| Formal education | During the last 2 years, has this person participated in any activity related with gaining new professional qualifications or other skills?  If yes, specify the type (up to three types) of educational activity. | | Yes; No +  11 nursery, public kindergarten  12 nursery, private kindergarten  21 education in a public primary and lower secondary school  22 education in a private primary and lower secondary school  30 education in a basic vocational school, vocational traineeship  41 education in a public general secondary school  42 education in a private general secondary school  51 education in a public vocational secondary school  52 education in a private vocational secondary school  61 education in a public post-secondary school  62 education in a private post-secondary school  71 public higher education school  72 private higher education school  81 postgraduate studies in a public higher education school  82 postgraduate studies in a private higher education school  83 PhD studies in a public higher education school  84 PhD studies in a private higher education school  90 training courses and trainings financed by the employer  91 training courses and trainings financed from the Labour Fund  92 training courses and trainings financed from the European Social Fund  93 training courses and trainings financed with own resources of the household  94 other forms of mastering skills (such as driving lessons, learning how to play an instrument,  learning a foreign language)  98 I do not know.  Recoded as 1 if any type >20 and <80 was chosen. |
| New skills | In the last year, have you gained new qualifications or skills in order to have a higher salary? | | Yes; No |
| No need for books | In the last year, has any of the members of your household been unable to afford: (The answer “Not applicable” specifies the lack of the given need.):  purchase of a book | | 1. Yes; 2. No; 3. Not applicable  Recoded as a binary variable (3 as 1). |
| No books | How many books (approximately) are there at your home (excluding school books and manuals)? | | 1 none  2 up to 25 volumes  3 26-50 volumes  4 51-100 volumes  5 101-500 volumes  6 more than 500 volumes  Recoded as a binary variable (1 as 1). |
| Large city (>500k) | Dummy for cities with more than 500,000 inhabitants (pre-coded) | | |
| Big city (200-500k) | Dummy for cities with more than 200,000 but less than 500,000 inhabitants (pre-coded) | | |
| Medium city (100-200k) | Dummy for cities with more than 100,000 but less than 200,000 inhabitants (pre-coded) | | |
| Small city (20-100k) | Dummy for cities with more than 20,000 but less than 100,000 inhabitants (pre-coded) | | |
| Town (<20k) | Dummy for cities with less than 20,000 inhabitants (pre-coded) | | |
| Rural | Dummy for rural areas (pre-coded) | | |
| dolnośląskie  kujawsko-pomorskie  lubelskie  lubuskie  łódzkie  małopolskie  mazowieckie  opolskie  podkarpackie  podlaskie  pomorskie  śląskie  świętokrzyskie  warmińsko-mazurskie  wielkopolskie  zachodniopomorskie | Dummies for particular voivodships (NTS-2 areas). | | |
| Disability | Disability | 1 for the persons who have a valid certificate from the Social Insurance Institution (ZUS);  2 for the persons who have a valid certificate from the Disability Evaluation Board at the Poviat Centre  of Family Support (ZOoN at PCPR);  3 for the persons who have a valid certificate from the Social Insurance Institution and ZOoN at PCPR;  4 for the persons who have stated that due to disability or disease they have completely or partly  limited ability to perform such activities as learning, working or taking care of own household but they  do not have a certificate from the medical board;  5 disability of children aged below 16;  0 other cases;  8 not applicable (the person is not a disabled person)  Recoded as a binary variable (0-5 as 1) | |
| Marital status | Marital status | 1 unmarried  2 married  3 widow(er)  4 divorced  5 legally separated (based on a court decision)  6 practically separated (the spouses do not live together without a court decision).  Recoded as binary (2 as 1) | |
| Number of children (0-4) | Number of children being up to 4 years old in the same household (calculated by matching household ID number). | | |
| Number of children (5-9) | Number of children being over 5 years old but under 10 in the same household (calculated by matching household ID number). | | |
| Number of teens (10-14) | Number of adolescents being over 10 years old but under 15 in the same household (calculated by matching household ID number). | | |
| Number of teens (15-19) | Number of adolescents being over 15 years old but under 19 in the same household (calculated by matching household ID number). | | |
| Number of adults | Number of other adults - people at age of at least 20^[[1]](#footnote-1)^ - in the same household (calculated by matching household ID number). | | |
| Number of children (0-1) | Number of children being up to 1 year old in the same household (calculated by matching household ID number). | | |
| Household ratio of adults sport activity | Number of children and adolescents engaged in sport activity, up to 19 years old over the total number of children and adolescents, up to 19 years old, who have filled in the individual questionnaire, living in the same household (calculated by matching household ID number; when no children or adolescents participated in the study, value = 0). | | |
| Household ratio of u20 sport activity | Number of other adults engaged in sport activity, at least 20 years old over the total number of other adults, at least 20 years old, who have filled in the individual questionnaire, living in the same household (calculated by matching household ID number; when no other adults participated in the study, value = 0). | | |
| Permanent employment | What is the type of work this person performs at his/her main job? | 1. based on an employment contract for a specified period of time (apart from the contracts listed  below, being non-standard forms of employment (6-11), and for a period longer than one year)  2. based on an employment contract for an unspecified period of time  3. self-employed entrepreneur hiring employers  4. self-employed  5. helping in a family business without pay  6. temporary job (based on fixed-term employment contracts, such as replacement contracts,  contracts for specific work)  7. other short-term contracts (such as summer traineeships, employment contracts for a period shorter  than one year)  8. trial period employment  9. paid employment on the basis of a civil law contract (contract of mandate, contract for specific  work)  10. paid employment without a formal contract or with an oral agreement  11. other  Recoded as binary (2 as 1) | |
| Entrepreneur | As above | Answers as above – recoded 3 as 1 | |
| Self-employed | As above | Answers as above – recoded 4 as 1 | |
| Inactive | During the last 4 weeks, has this person been seeking a job or a different job? | 1 YES and I am currently unemployed  2 YES and I am currently employed  3 NO and I am currently unemployed but I have already found a job  4 NO and I am currently unemployed  5 NO and I am currently employed  Recoded as binary (4 as 1) | |
| Unemployed | During the last 4 weeks, has this person been seeking a job or a different job? | 1 YES and I am currently unemployed  2 YES and I am currently employed  3 NO and I am currently unemployed but I have already found a job  4 NO and I am currently unemployed  5 NO and I am currently employed  Recoded as binary (1 and 3 as 1) | |
| Retired (not working) | Based on Source of income: main and additional | 11 permanent paid employment in the public sector 12 permanent paid employment in the private sector 13 temporary paid employment in the public sector 14 temporary paid employment in the private sector 15 use of an agricultural holding 16 helping in an agricultural holding 17 employer outside an individual holding in agriculture 18 permanent work for one’s own account (also self-employment) 19 temporary work for one’s own account 20 Helping in work for one’s own account 21 old age pension (apart from the agricultural social insurance system) 22 old age pensions for individual farmers (under insurance in the Agricultural Social Insurance Fund, KRUS) 23 disability pensions 24 family pensions 25 maternity benefits 26 unemployment benefits 27 other benefits from the Labour Fund 28 allowance for persons on child care leaves (former child care benefits) 29 other social insurance benefits (such as child birth allowance, funeral allowance, sickness allowance) 30 family benefits and allowance in accordance with the Act on Family Benefits of 2003, as amended, housing allowance 31 social assistance benefits 32 other social assistance benefits (such as benefits for persons brining up children, special purpose benefits and extraordinary benefits) 33 children maintenance 34 other income of a social benefit nature (including scholarships)  35 income from own property (interest, dividends, etc.) 36 income from the rental of a house, apartment or garage 37 foreign old age and disability pensions 38 benefits under a voluntary sickness and accident insurance system  39 compensation under other insurance schemes 40 donations, maintenance from private persons 41 other income 42 other revenues (sale of property, savings, credits) 43 being supported by other household members  Recoded as binary variable ((main = (21 or 22) and (additional >20 or empty)) as 1) | |
| Retired working | As above | Recoded as binary variable ((additional = (21 or 22) or ((main = (21 or 22) and (additional < 20) and nonempty)) as 1 | |
| Inflexible worktime | Is it possible at your main job to change the time you start or finish your work day | Yes; No | |
| Longer breaks in work impossible | Is it possible at your main job to leave your work for at least an hour | Yes; No | |
| Inflexible work (place) | Is it possible at your main job to change the time you start or finish your work day | Yes; No | |
| Working full time | Is it possible at your main job to perform some of your professional duties at home | Yes; No | |
| Changed job for a better one | In the last year, have you started a better paid or an additional job? | Yes; No | |
| Too little time with the child | In the recent months you have spent too little time with your child | 1. Often; 2. Once or twice; 3. Never; 4. Not applicable. Recoded as binary (1 as 1). | |
| Overburdened with work duties | In the recent months you have felt overburdened with work duties which you have been unable to cope with | 1. Often; 2. Once or twice; 3. Never; 4. Not applicable. Recoded as binary (1 as 1). | |
| Armed forces  Managers and officials  Professionals  Technicians  Clerical support  Service and sales  Farmers  Craft workers  Plant/machine operators  Elementary occupations | Dummy for occupational groups (according to the ISCO classification) – based on current occupation or at the last place of work (pre-coded) | | |
| Higher education of father | What was the educational attainment of your father (or main guardian) when you were 14? | 1 primary not completed  2 primary  3 vocational  4 secondary not completed  5 secondary vocational  6 secondary general  7 higher not completed (including post-secondary)  8 higher  9 I do not know.  Recoded as binary (7 and 8 as 1). | |
| General trust | In general, do you believe that most people can be trusted or are you of the opinion that one can never be too  careful with people? | 1 most people can be trusted  2 one cannot be too careful in dealing with people  3 it is hard to say  Recoded as binary (1 as 1). | |
| Trust towards Parliament | Do you trust Sejm? | 1 yes  2 no  3 I have no opinion  Recoded as binary (1 as 1). | |
| Trust towards banks | Do you trust commercial banks? | As above. | |
| Trust towards the president | Do you trust President? | As above. | |
| Trust towards stock exchange | Do you trust stock exchange? | As above. | |
| Trust towards the NBP | Do you trust National Bank of Poland? | As above. | |
| Trust towards the family | Do you trust own family members? | As above. | |
| Trust towards the neighbours | Do you trust neighbours? | As above. | |
| Trust towards nonstate pension funds | Do you trust commercial banks? | As above. | |
| Trust towards courts | Do you trust courts? | As above. | |
| Trust towards the European Parliament | Do you trust European Parliament? | As above. | |
| Trust towards the police | Do you trust police? | As above. | |
| Trust towards the government | Do you trust government? | As above. | |
| Trust towards the state social security system | Do you trust Social Insurance Institution (ZUS)? | As above. | |
| Income | During the last three months, your own (personal) monthly net income (less taxes) has on average amounted to: | Open-ended (integer). (Recoded as 0 when unemployed or inactive) | |
| Mobile phone | Does he/she have a mobile phone, a smartphone or a PDA? | 1 Yes, a mobile phone,  2 Yes, a smartphone or a PDA,  3 – both devices,  4 No, none of these  Recoded as binary (1,2 and 3 as 1). | |
| No wasching machine | Does your household or any of its members possess the following goods? It does not matter whether such goods are owned, leased or made available in any other manner (provide the answers in the column "Does the household possess?”). If the household does not possess a specific item, please specify (provide the answer in the column ―”If not, is it due to financial reasons?”) whether this is due to financial reasons (answer ―YES) or any other reasons, for example such item is redundant (answer ―NO):  automatic washing machine | Recoded as binary (Yes to the question ”If not, is it due to financial reasons?” as 1). | |
| No paid TV | Does your household or any of its members possess the following goods? It does not matter whether such goods are owned, leased or made available in any other manner (provide the answers in the column "Does the household possess?”). If the household does not possess a specific item, please specify (provide the answer in the column ―”If not, is it due to financial reasons?”) whether this is due to financial reasons (answer ―YES) or any other reasons, for example such item is redundant (answer ―NO):  paid satellite or cable TV | Recoded as binary (Yes to the question ”If not, is it due to financial reasons?” as 1). | |
| No independent apartment | Does your household share your dwelling with any other household? | Yes; No | |
| Size of the living space | What is the total useable floor space of the dwelling your household lives in, in full square metres? | Open-ended (integer) | |
| Social assistance | Does your household receive any external assistance? | Yes; No | |
| Health benefits | In the last year, have you used the services of healthcare units paid for by the employer (under a medical services plan or health insurance)? | Yes; No | |
| No decrease in energy to work | Read the four statements in each point carefully and then choose one  that describes best your feelings and beliefs during the last month | 0. I have as much energy as ever to work.  1. I have less energy than I used to have.  2. I don’t have enough energy to do much.  3. I don’t have enough energy to do anything. Recoded as a binary variable (0 recoded as 1). | |

Based on Social Diagnosis, questionnaires. www.diagnoza.com [downloaded 19.03.2017].

**Table B.** **Variables based on Statistics Poland and Orliki databases.**

| **Variable** | **Definition** |
| --- | --- |
| Number of stadiums 2011 | Number of stadiums (multi-purpose, athletics, football, speedway, rugby) per capita in a given subregion (NTS-3 level), calculated based on number of entries in the KFT-1, KFT-OB/a, KFT-OB/b surveys conducted in 2014 and on declared year of construction of the object |
| Number of fields 2011 | Number of stadiums (both full-size and not full-size; multi-purpose, football, rugby, hockey, baseball and softball, basketball, handball, volleyball, beach volleyball, beach handball) per capita in a given subregion (NTS-3 level), calculated based on number of entries in the KFT-1, KFT-OB/a, KFT-OB/b surveys conducted in 2014 and on declared year of construction of the object |
| Number of indoor arenas 2011 | Number of stadiums (multi-purpose, athletics, football, speedway, rugby) per capita in a given subregion (NTS-3 level), calculated based on number of entries in the KFT-1, KFT-OB/a, KFT-OB/b surveys conducted in 2014 and on declared year of construction of the object |
| Number of gyms 2011 | Number of gyms (of different sizes) per capita in a given subregion (NTS-3 level), calculated based on number of entries in the KFT-1, KFT-OB/a, KFT-OB/b surveys conducted in 2014 and on declared year of construction of the object |
| Number of courts 2011 | Number of courts (open & closed tennis courts, squash courts) per capita in a given subregion (NTS-3 level), calculated based on number of entries in the KFT-1, KFT-OB/a, KFT-OB/b surveys conducted in 2014 and on declared year of construction of the object |
| Number of golf fields 2011 | Number of golf fields per capita in a given subregion (NTS-3 level), calculated based on number of entries in the KFT-1, KFT-OB/a, KFT-OB/b surveys conducted in 2014 and on declared year of construction of the object |
| Number of swimming pools 2011 | Number of swimming pools (open & closed) per capita in a given subregion (NTS-3 level), calculated based on number of entries in the KFT-1, KFT-OB/a, KFT-OB/b surveys conducted in 2014 and on declared year of construction of the object |
| Number of horse tracks 2011 | Number of horse tracks (hippodromes, riding tracks) per capita in a given subregion (NTS-3 level), calculated based on number of entries in the KFT-1, KFT-OB/a, KFT-OB/b surveys conducted in 2014 and on declared year of construction of the object |
| Number of shooting 2011 | Number of shooting facilities (archery tracks, shooting ranges) per capita in a given subregion (NTS-3 level), calculated based on number of entries in the KFT-1, KFT-OB/a, KFT-OB/b surveys conducted in 2014 and on declared year of construction of the object |
| Number of winter 2011 | Number of winter facilities (toboggan runs, skating tracks, open & closed artificial ice rinks, ski runner trails, cross-country ski runs, ski jumps) per capita in a given subregion (NTS-3 level), calculated based on number of entries in the KFT-1, KFT-OB/a, KFT-OB/b surveys conducted in 2014 and on declared year of construction of the object |
| Number of motorsports 2011 | Number of racing tracks (water tracks – canoe, rowing, regatta, motorcycle tracks, kart tracks, car tracks) per capita in a given subregion (NTS-3 level), calculated based on number of entries in the KFT-1, KFT-OB/a, KFT-OB/b surveys conducted in 2014 and on declared year of construction of the object |
| Number of outdoor 2011 | Number of outdoor facilities (roller ski trails, bike paths, skateparks, outdoor gyms, running tracks) per capita in a given subregion (NTS-3 level), calculated based on number of entries in the KFT-1, KFT-OB/a, KFT-OB/b surveys conducted in 2014 and on declared year of construction of the object. |
| Number of Orliki 2010 | Number of the Orliki objects per capita in a given subregion (NTS-3 level), calculated based on number of entries in the registry of the object for the particular year. |

Based on Statistics Poland. (2014). Surveys on sports facilities - results of KFT-OB/a (municipalities), KFT-OB/b (external administrators), KFT-1 (sport clubs) surveys. Statistics Poland.] and Biernat, E., Piątkowska, M., Zembura, P., & Gołdys, A. (2017). Problem zarządzania orlikami z perspektywy animatorów z gmin wiejskich i miejskich [Challenges of management of Orlik pitches in the perspective of the animators from municipal and rural communities]. Przedsiębiorczość i zarządzania, 18(8), 429–444.

1. Even though 18 is age of maturity in Poland, 19 has been chosen as the highest age for ‘non-adults’, since most of the high schools students graduate during the summer in a year in which they are 19 years old. [↑](#footnote-ref-1)
