# Supplementary material for: Impact of sports activity on Polish adults: Self-reported health, social capital & attitudes
Source: PLoS One. 2019 Dec 19;14(12):e0226812. doi: 10.1371/journal.pone.0226812 (PMC6922371; doi:10.1371/journal.pone.0226812)
Supplement: S2 Appendix — (DOCX) [file pone.0226812.s002.docx]

# S2 Appendix. Descriptive statistics.

Table A. Confounding variables – descriptive statistics for women.

|  | **count** | **mean** | **median** | **sd** | **min** | **max** |
| --- | --- | --- | --- | --- | --- | --- |
| Large city (>500k) | 6115 | 0.075 | dummy variable | | | |
| Big city (200-500k) | 6115 | 0.087 | dummy variable | | | |
| Medium city (100-200k) | 6115 | 0.065 | dummy variable | | | |
| Small city (20-100k) | 6115 | 0.19 | dummy variable | | | |
| Town (<20k) | 6115 | 0.13 | dummy variable | | | |
| dolnośląskie | 6115 | 0.067 | dummy variable | | | |
| kujawsko-pomorskie | 6115 | 0.053 | dummy variable | | | |
| lubelskie | 6115 | 0.071 | dummy variable | | | |
| lubuskie | 6115 | 0.033 | dummy variable | | | |
| łódzkie | 6115 | 0.069 | dummy variable | | | |
| małopolskie | 6115 | 0.073 | dummy variable | | | |
| mazowieckie | 6115 | 0.11 | dummy variable | | | |
| opolskie | 6115 | 0.034 | dummy variable | | | |
| podkarpackie | 6115 | 0.066 | dummy variable | | | |
| podlaskie | 6115 | 0.045 | dummy variable | | | |
| pomorskie | 6115 | 0.058 | dummy variable | | | |
| śląskie | 6115 | 0.10 | dummy variable | | | |
| świętokrzyskie | 6115 | 0.046 | dummy variable | | | |
| warmińsko-mazurskie | 6115 | 0.050 | dummy variable | | | |
| wielkopolskie | 6115 | 0.072 | dummy variable | | | |
| zachodniopomorskie | 6115 | 0.045 | dummy variable | | | |
| Married 2011 | 6115 | 0.73 | dummy variable | | | |
| Married 2013 | 6115 | 0.72 | dummy variable | | | |
| Number of children (0-4) 2011 | 6115 | 0.21 | 0 | 0.49 | 0 | 3 |
| Number of children (5-9) 2011 | 6115 | 0.21 | 0 | 0.49 | 0 | 4 |
| Number of teens (10-14) 2011 | 6115 | 0.24 | 0 | 0.53 | 0 | 5 |
| Number of teens (15-19) 2011 | 6115 | 0.29 | 0 | 0.59 | 0 | 4 |
| Number of adults 2011 | 6115 | 3.03 | 3 | 1.39 | 1 | 12 |
| Number of children (0-1) 2013 | 6115 | 0.039 | 0 | 0.20 | 0 | 2 |
| Higher education, father 2011 | 6115 | 0.054 | dummy variable | | | |
| Educated her/himself 2011 | 6115 | 0.10 | dummy variable | | | |
| Formal education 2011 | 6115 | 0.019 | dummy variable | | | |
| Too little time with the child 2011 | 6115 | 0.028 | dummy variable | | | |
| Overburdened with work duties 2011 | 6115 | 0.049 | dummy variable | | | |
| Number of social meetings 2011 | 6115 | 1.41 | 1 | 2.13 | 0 | 30 |
| Number of entertainment 2011 | 6115 | 0.28 | 0 | 0.74 | 0 | 10 |
| Number of restaurants 2011 | 6115 | 0.59 | 0 | 1.43 | 0 | 30 |
| Time using computer 2011 | 6115 | 7.46 | 2 | 12.8 | 0 | 90 |
| Number of cigarettes 2011 | 6115 | 3.22 | 0 | 6.61 | 0 | 60 |
| Too much alcohol 2010 | 6115 | 0.020 | dummy variable | | | |
| Drugs/designer drugs 2011 | 6115 | 0.0011 | dummy variable | | | |
| New skills 2011 | 6115 | 0.11 | dummy variable | | | |
| Age 2011 | 6115 | 46.8 | 48 | 11.3 | 25 | 64 |
| BMI 2011 | 6115 | 25.8 | 25.2 | 4.78 | 14.7 | 59.9 |
| Dissatisfaction with health 2011 | 6115 | 3.03 | 3 | 1.22 | 1 | 7 |
| Physical problems 2011 | 6115 | 0.65 | dummy variable | | | |
| Health problems 2011 | 6115 | 0.59 | dummy variable | | | |
| Rare suicidal thoughts 2011 | 6115 | 0.094 | dummy variable | | | |
| Often suicidal thoughts 2011 | 6115 | 0.030 | dummy variable | | | |
| Seriously ill 2010 | 6115 | 0.13 | dummy variable | | | |
| Disability 2011 | 6115 | 0.11 | dummy variable | | | |
| Problems with sleeping 2011 | 6115 | 0.092 | dummy variable | | | |
| Some worries about digestive system 2011 | 6115 | 0.30 | dummy variable | | | |
| Constant worries about health 2011 | 6115 | 0.13 | dummy variable | | | |
| Losing interest in sex 2011 | 6115 | 0.63 | dummy variable | | | |
| Headaches (<1/2) 2011 | 6115 | 0.43 | dummy variable | | | |
| Headaches (>1/2) 2011 | 6115 | 0.071 | dummy variable | | | |
| Stomach pains (<1/2) 2011 | 6115 | 0.35 | dummy variable | | | |
| Stomach pains (>1/2) 2011 | 6115 | 0.060 | dummy variable | | | |
| Pain in neck or arm muscles (<1/2) 2011 | 6115 | 0.40 | dummy variable | | | |
| Pain in neck or arm muscles (>1/2) 2011 | 6115 | 0.11 | dummy variable | | | |
| Chest or heart pains (<1/2) 2011 | 6115 | 0.21 | dummy variable | | | |
| Chest or heart pains (>1/2) 2011 | 6115 | 0.033 | dummy variable | | | |
| Dry mouth or throat (<1/2) 2011 | 6115 | 0.22 | dummy variable | | | |
| Dry mouth or throat (>1/2) 2011 | 6115 | 0.047 | dummy variable | | | |
| Sweating (<1/2) 2011 | 6115 | 0.25 | dummy variable | | | |
| Sweating (>1/2) 2011 | 6115 | 0.066 | dummy variable | | | |
| Shortness of breath (<1/2) 2011 | 6115 | 0.19 | dummy variable | | | |
| Shortness of breath (>1/2) 2011 | 6115 | 0.038 | dummy variable | | | |
| Body pains (<1/2) 2011 | 6115 | 0.37 | dummy variable | | | |
| Body pains (>1/2) 2011 | 6115 | 0.076 | dummy variable | | | |
| Palpitation (<1/2) 2011 | 6115 | 0.20 | dummy variable | | | |
| Palpitation (>1/2) 2011 | 6115 | 0.036 | dummy variable | | | |
| Shivers or convulsions (<1/2) 2011 | 6115 | 0.069 | dummy variable | | | |
| Shivers or convulsions (>1/2) 2011 | 6115 | 0.011 | dummy variable | | | |
| Pressure on bladder (<1/2) 2011 | 6115 | 0.18 | dummy variable | | | |
| Pressure on bladder (>1/2) 2011 | 6115 | 0.048 | dummy variable | | | |
| Tiredness (<1/2) 2011 | 6115 | 0.38 | dummy variable | | | |
| Tiredness (>1/2) 2011 | 6115 | 0.074 | dummy variable | | | |
| Constipation (<1/2) 2011 | 6115 | 0.17 | dummy variable | | | |
| Constipation (>1/2) 2011 | 6115 | 0.041 | dummy variable | | | |
| Nosebleeds (<1/2) 2011 | 6115 | 0.047 | dummy variable | | | |
| Nosebleeds (>1/2) 2011 | 6115 | 0.0074 | dummy variable | | | |
| Blood pressure (<1/2) 2011 | 6115 | 0.20 | dummy variable | | | |
| Blood pressure (>1/2) 2011 | 6115 | 0.059 | dummy variable | | | |
| Years of education 2011 | 6115 | 12.4 | 12 | 3.08 | 1 | 25 |
| Higher Education 2011 | 6115 | 0.20 | dummy variable | | | |
| English 2011 | 6115 | 0.11 | dummy variable | | | |
| German 2011 | 6115 | 0.047 | dummy variable | | | |
| French 2011 | 6115 | 0.0095 | dummy variable | | | |
| Russian 2011 | 6115 | 0.10 | dummy variable | | | |
| Spanish 2011 | 6115 | 0.0023 | dummy variable | | | |
| other language 2011 | 6115 | 0.011 | dummy variable | | | |
| No need for books 2011 | 6115 | 0.11 | dummy variable | | | |
| No books 2011 | 6115 | 0.078 | dummy variable | | | |
| Afraid of neighbourhood 2011 | 6115 | 0.060 | dummy variable | | | |
| Voted in 2010 | 6115 | 0.73 | dummy variable | | | |
| Number of social meetings 2011 | 6115 | 3.00 | 4 | 2.78 | 0 | 30 |
| Public meetings 2011 | 6115 | 0.26 | dummy variable | | | |
| Member of organisations 2011 | 6115 | 0.19 | 0 | 0.48 | 0 | 3 |
| Fullfiling roles in organisations 2011 | 6115 | 0.051 | dummy variable | | | |
| Voluntary activities 2011 | 6115 | 0.18 | dummy variable | | | |
| Number of friends met 2011 | 6115 | 5.00 | 4 | 4.85 | 0 | 60 |
| Number of acquaintances met 2011 | 6115 | 6.14 | 4 | 7.02 | 0 | 70 |
| Number of family members met 2011 | 6115 | 8.95 | 8 | 6.97 | 0 | 60 |
| Number of friends 2011 | 6115 | 6.49 | 5 | 6.41 | 0 | 99 |
| Work for local society 2011 | 6115 | 0.17 | dummy variable | | | |
| General trust 2011 | 6115 | 0.14 | dummy variable | | | |
| Trust towards banks 2011 | 6115 | 0.19 | dummy variable | | | |
| Trust towards Parliament 2011 | 6115 | 0.14 | dummy variable | | | |
| Trust towards the president 2011 | 6115 | 0.29 | dummy variable | | | |
| Trust towards stock exchange 2011 | 6115 | 0.052 | dummy variable | | | |
| Trust towards the NBP 2011 | 6115 | 0.49 | dummy variable | | | |
| Trust towards the family 2011 | 6115 | 0.91 | dummy variable | | | |
| Trust towards the neighbours 2011 | 6115 | 0.60 | dummy variable | | | |
| Trust towards nonstate pension funds 2011 | 6115 | 0.099 | dummy variable | | | |
| Trust towards courts 2011 | 6115 | 0.33 | dummy variable | | | |
| Trust towards the European Parliament 2011 | 6115 | 0.21 | dummy variable | | | |
| Trust towards the police 2011 | 6115 | 0.48 | dummy variable | | | |
| Trust towards the government 2011 | 6115 | 0.17 | dummy variable | | | |
| Trust towards the state social security system 2011 | 6115 | 0.25 | dummy variable | | | |
| Lust for life2011 | 6115 | 8.55 | 9 | 1.79 | 1 | 10 |
| Achieving goals > fun 2011 | 6115 | 0.62 | dummy variable | | | |
| Success depended on her/himself 2011 | 6115 | 0.68 | dummy variable | | | |
| Belief in democracy 2011 | 6115 | 0.25 | dummy variable | | | |
| Supports Law and Justice 2011 | 6115 | 0.14 | dummy variable | | | |
| Supports no political party 2011 | 6115 | 0.40 | dummy variable | | | |
| Fun is most important 2011 | 6115 | 0.62 | dummy variable | | | |
| Entire life delightful or pleasing 2011 | 6115 | 0.42 | dummy variable | | | |
| Money - 1 of 3 most important things in life 2011 | 6115 | 0.23 | dummy variable | | | |
| No decrease in energy to work 2011 | 6115 | 0.68 | dummy variable | | | |
| Reforms in Poland successful 2011 | 6115 | 0.13 | dummy variable | | | |
| Mobile phone 2011 | 6115 | 0.87 | dummy variable | | | |
| Income 2011 | 6115 | 3532.3 | 3000 | 2318.9 | 100 | 44000 |
| Health benefits 2011 | 6115 | 0.044 | dummy variable | | | |
| No wasching machine 2011 | 6115 | 0.042 | dummy variable | | | |
| No paid TV 2011 | 6115 | 0.13 | dummy variable | | | |
| No independent apartment 2011 | 6115 | 0.032 | dummy variable | | | |
| Size of the living space 2011 | 6115 | 85.9 | 70 | 60.7 | 5 | 901 |
| Social assistance 2011 | 6115 | 0.10 | dummy variable | | | |
| Entrepreneur 2011 | 6115 | 0.0096 | dummy variable | | | |
| Self-employed 2011 | 6115 | 0.086 | dummy variable | | | |
| Unemployed 2011 | 6115 | 0.065 | dummy variable | | | |
| Inactive (not retired) 2011 | 6115 | 0.22 | dummy variable | | | |
| Retired (not working) 2011 | 6115 | 0.16 | dummy variable | | | |
| Retired working 2011 | 6115 | 0.023 | dummy variable | | | |
| Inflexible worktime 2011 | 6115 | 0.29 | dummy variable | | | |
| Longer breaks in work impossible 2011 | 6115 | 0.25 | dummy variable | | | |
| Inflexible work (place) 2011 | 6115 | 0.38 | dummy variable | | | |
| Permanent employment 2011 | 6115 | 0.32 | dummy variable | | | |
| Working full time 2011 | 6115 | 0.50 | dummy variable | | | |
| Armed forces 2011 | 6115 | 0.070 | dummy variable | | | |
| Managers and officials 2011 | 6115 | 0.030 | dummy variable | | | |
| Professionals 2011 | 6115 | 0.15 | dummy variable | | | |
| Technicians 2011 | 6115 | 0.10 | dummy variable | | | |
| Clerical support 2011 | 6115 | 0.085 | dummy variable | | | |
| Service and sales 2011 | 6115 | 0.18 | dummy variable | | | |
| Farmers 2011 | 6115 | 0.14 | dummy variable | | | |
| Craft workers 2011 | 6115 | 0.089 | dummy variable | | | |
| Plant/machine operators 2011 | 6115 | 0.027 | dummy variable | | | |
| Elementary occupations 2011 | 6115 | 0.13 | dummy variable | | | |
| Household ratio of adults sport activity 2011 | 6115 | 0.30 | dummy variable | | | |
| Household ratio of u20 sport activity 2011 | 6115 | 0.11 | dummy variable | | | |
| Number of stadiums 2011 | 6115 | 5.64 | 5.27 | 3.29 | 0.71 | 21.3 |
| Number of fields 2011 | 6115 | 2.21 | 2.08 | 1.35 | 0.27 | 7.58 |
| Number of indoor arenas 2011 | 6115 | 1.67 | 1.67 | 0.71 | 0.32 | 3.54 |
| Number of gyms 2011 | 6115 | 1.60 | 1.48 | 0.86 | 0.20 | 5.01 |
| Number of courts 2011 | 6115 | 3.38 | 3.39 | 1.46 | 0.80 | 7.96 |
| Number of golf fields 2011 | 6115 | 2.25 | 0 | 7.13 | 0 | 37.1 |
| Number of swimming pools 2011 | 6115 | 1.46 | 1.42 | 0.80 | 0.31 | 3.86 |
| Number of horse tracks 2011 | 6115 | 1.26 | 0 | 1.95 | 0 | 8.90 |
| Number of shooting 2011 | 6115 | 3.64 | 2.64 | 4.10 | 0 | 25.5 |
| Number of winter 2011 | 6115 | 5.46 | 3.82 | 7.26 | 0 | 51.0 |
| Number of motorsports 2011 | 6115 | 1.58 | 1.40 | 2.08 | 0 | 12.6 |
| Number of outdoor 2011 | 6115 | 4.09 | 3.62 | 2.63 | 0.32 | 12.1 |
| Number of Orliki 2010 | 6115 | 4.92 | 4.79 | 2.29 | 0.48 | 10.8 |

The table presents ratios of ‘1’ values for dummy variables and means, medians, standard deviations, minimum values and maximum values for continuous and categorical variables.

Table B. Confounding variables – descriptive statistics for men.

|  | **count** | **mean/ratio** | **median** | **sd** | **min** | **max** |
| --- | --- | --- | --- | --- | --- | --- |
| Large city (>500k) | 4746 | 0.068 | dummy variable | | | |
| Big city (200-500k) | 4746 | 0.074 | dummy variable | | | |
| Medium city (100-200k) | 4746 | 0.055 | dummy variable | | | |
| Small city (20-100k) | 4746 | 0.17 | dummy variable | | | |
| Town (<20k) | 4746 | 0.12 | dummy variable | | | |
| dolnośląskie | 4746 | 0.059 | dummy variable | | | |
| kujawsko-pomorskie | 4746 | 0.055 | dummy variable | | | |
| lubelskie | 4746 | 0.069 | dummy variable | | | |
| lubuskie | 4746 | 0.034 | dummy variable | | | |
| łódzkie | 4746 | 0.073 | dummy variable | | | |
| małopolskie | 4746 | 0.072 | dummy variable | | | |
| mazowieckie | 4746 | 0.12 | dummy variable | | | |
| opolskie | 4746 | 0.032 | dummy variable | | | |
| podkarpackie | 4746 | 0.064 | dummy variable | | | |
| podlaskie | 4746 | 0.043 | dummy variable | | | |
| pomorskie | 4746 | 0.057 | dummy variable | | | |
| śląskie | 4746 | 0.095 | dummy variable | | | |
| świętokrzyskie | 4746 | 0.051 | dummy variable | | | |
| warmińsko-mazurskie | 4746 | 0.056 | dummy variable | | | |
| wielkopolskie | 4746 | 0.074 | dummy variable | | | |
| zachodniopomorskie | 4746 | 0.043 | dummy variable | | | |
| Married 2011 | 4746 | 0.75 | dummy variable | | | |
| Married 2013 | 4746 | 0.76 | dummy variable | | | |
| Number of children (0-4) 2011 | 4746 | 0.21 | 0 | 0.49 | 0 | 3 |
| Number of children (5-9) 2011 | 4746 | 0.20 | 0 | 0.49 | 0 | 4 |
| Number of teens (10-14) 2011 | 4746 | 0.23 | 0 | 0.53 | 0 | 5 |
| Number of teens (15-19) 2011 | 4746 | 0.28 | 0 | 0.58 | 0 | 4 |
| Number of adults 2011 | 4746 | 3.19 | 3 | 1.41 | 1 | 12 |
| Number of children (0-1) 2013 | 4746 | 0.039 | 0 | 0.20 | 0 | 2 |
| Higher education, father 2011 | 4746 | 0.047 | dummy variable | | | |
| Educated her/himself 2011 | 4746 | 0.095 | dummy variable | | | |
| Formal education 2011 | 4746 | 0.014 | dummy variable | | | |
| Too little time with the child 2011 | 4746 | 0.060 | dummy variable | | | |
| Overburdened with work duties 2011 | 4746 | 0.055 | dummy variable | | | |
| Number of social meetings 2011 | 4746 | 1.46 | 1 | 2.46 | 0 | 30 |
| Number of entertainment 2011 | 4746 | 0.24 | 0 | 0.87 | 0 | 22 |
| Number of restaurants 2011 | 4746 | 0.81 | 0 | 2.13 | 0 | 30 |
| Time using computer 2011 | 4746 | 7.64 | 1 | 13.5 | 0 | 99 |
| Number of cigarettes 2011 | 4746 | 7.04 | 0 | 10.00 | 0 | 70 |
| Too much alcohol 2010 | 4746 | 0.13 | dummy variable | | | |
| Drugs/designer drugs 2011 | 4746 | 0.0088 | dummy variable | | | |
| New skills 2011 | 4746 | 0.11 | dummy variable | | | |
| Age 2011 | 4746 | 46.3 | 48 | 11.3 | 25 | 64 |
| BMI 2011 | 4746 | 27.2 | 26.7 | 4.15 | 16.3 | 76.1 |
| Dissatisfaction with health 2011 | 4746 | 2.87 | 3 | 1.22 | 1 | 7 |
| Physical problems 2011 | 4746 | 0.62 | dummy variable | | | |
| Health problems 2011 | 4746 | 0.54 | dummy variable | | | |
| Rare suicidal thoughts 2011 | 4746 | 0.082 | dummy variable | | | |
| Often suicidal thoughts 2011 | 4746 | 0.025 | dummy variable | | | |
| Seriously ill 2010 | 4746 | 0.11 | dummy variable | | | |
| Disability 2011 | 4746 | 0.14 | dummy variable | | | |
| Problems with sleeping 2011 | 4746 | 0.084 | dummy variable | | | |
| Some worries about digestive system 2011 | 4746 | 0.24 | dummy variable | | | |
| Constant worries about health 2011 | 4746 | 0.11 | dummy variable | | | |
| Losing interest in sex 2011 | 4746 | 0.38 | dummy variable | | | |
| Headaches (<1/2) 2011 | 4746 | 0.29 | dummy variable | | | |
| Headaches (>1/2) 2011 | 4746 | 0.041 | dummy variable | | | |
| Stomach pains (<1/2) 2011 | 4746 | 0.24 | dummy variable | | | |
| Stomach pains (>1/2) 2011 | 4746 | 0.034 | dummy variable | | | |
| Pain in neck or arm muscles (<1/2) 2011 | 4746 | 0.39 | dummy variable | | | |
| Pain in neck or arm muscles (>1/2) 2011 | 4746 | 0.075 | dummy variable | | | |
| Chest or heart pains (<1/2) 2011 | 4746 | 0.21 | dummy variable | | | |
| Chest or heart pains (>1/2) 2011 | 4746 | 0.038 | dummy variable | | | |
| Dry mouth or throat (<1/2) 2011 | 4746 | 0.20 | dummy variable | | | |
| Dry mouth or throat (>1/2) 2011 | 4746 | 0.032 | dummy variable | | | |
| Sweating (<1/2) 2011 | 4746 | 0.16 | dummy variable | | | |
| Sweating (>1/2) 2011 | 4746 | 0.032 | dummy variable | | | |
| Shortness of breath (<1/2) 2011 | 4746 | 0.15 | dummy variable | | | |
| Shortness of breath (>1/2) 2011 | 4746 | 0.029 | dummy variable | | | |
| Body pains (<1/2) 2011 | 4746 | 0.37 | dummy variable | | | |
| Body pains (>1/2) 2011 | 4746 | 0.061 | dummy variable | | | |
| Palpitation (<1/2) 2011 | 4746 | 0.14 | dummy variable | | | |
| Palpitation (>1/2) 2011 | 4746 | 0.023 | dummy variable | | | |
| Shivers or convulsions (<1/2) 2011 | 4746 | 0.056 | dummy variable | | | |
| Shivers or convulsions (>1/2) 2011 | 4746 | 0.0063 | dummy variable | | | |
| Pressure on bladder (<1/2) 2011 | 4746 | 0.13 | dummy variable | | | |
| Pressure on bladder (>1/2) 2011 | 4746 | 0.036 | dummy variable | | | |
| Tiredness (<1/2) 2011 | 4746 | 0.31 | dummy variable | | | |
| Tiredness (>1/2) 2011 | 4746 | 0.049 | dummy variable | | | |
| Constipation (<1/2) 2011 | 4746 | 0.072 | dummy variable | | | |
| Constipation (>1/2) 2011 | 4746 | 0.0091 | dummy variable | | | |
| Nosebleeds (<1/2) 2011 | 4746 | 0.046 | dummy variable | | | |
| Nosebleeds (>1/2) 2011 | 4746 | 0.0063 | dummy variable | | | |
| Blood pressure (<1/2) 2011 | 4746 | 0.18 | dummy variable | | | |
| Blood pressure (>1/2) 2011 | 4746 | 0.047 | dummy variable | | | |
| Years of education 2011 | 4746 | 12.0 | 11 | 2.89 | 1 | 25 |
| Higher Education 2011 | 4746 | 0.14 | dummy variable | | | |
| English 2011 | 4746 | 0.10 | dummy variable | | | |
| German 2011 | 4746 | 0.044 | dummy variable | | | |
| French 2011 | 4746 | 0.0078 | dummy variable | | | |
| Russian 2011 | 4746 | 0.084 | dummy variable | | | |
| Spanish 2011 | 4746 | 0.0025 | dummy variable | | | |
| other language 2011 | 4746 | 0.0084 | dummy variable | | | |
| No need for books 2011 | 4746 | 0.14 | dummy variable | | | |
| No books 2011 | 4746 | 0.10 | dummy variable | | | |
| Afraid of neighbourhood 2011 | 4746 | 0.034 | dummy variable | | | |
| Voted in 2010 | 4746 | 0.71 | dummy variable | | | |
| Number of social meetings 2011 | 4746 | 2.19 | 2 | 2.47 | 0 | 60 |
| Public meetings 2011 | 4746 | 0.30 | dummy variable | | | |
| Member of organisations 2011 | 4746 | 0.21 | 0 | 0.54 | 0 | 3 |
| Fullfiling roles in organisations 2011 | 4746 | 0.068 | dummy variable | | | |
| Voluntary activities 2011 | 4746 | 0.25 | dummy variable | | | |
| Number of friends met 2011 | 4746 | 5.53 | 4 | 5.67 | 0 | 60 |
| Number of acquaintances met 2011 | 4746 | 6.82 | 4 | 8.15 | 0 | 80 |
| Number of family members met 2011 | 4746 | 8.51 | 6 | 7.07 | 0 | 60 |
| Number of friends 2011 | 4746 | 7.10 | 5 | 7.59 | 0 | 99 |
| Work for local society 2011 | 4746 | 0.20 | dummy variable | | | |
| General trust 2011 | 4746 | 0.14 | dummy variable | | | |
| Trust towards banks 2011 | 4746 | 0.21 | dummy variable | | | |
| Trust towards Parliament 2011 | 4746 | 0.14 | dummy variable | | | |
| Trust towards the president 2011 | 4746 | 0.31 | dummy variable | | | |
| Trust towards stock exchange 2011 | 4746 | 0.089 | dummy variable | | | |
| Trust towards the NBP 2011 | 4746 | 0.50 | dummy variable | | | |
| Trust towards the family 2011 | 4746 | 0.90 | dummy variable | | | |
| Trust towards the neighbours 2011 | 4746 | 0.59 | dummy variable | | | |
| Trust towards nonstate pension funds 2011 | 4746 | 0.10 | dummy variable | | | |
| Trust towards courts 2011 | 4746 | 0.32 | dummy variable | | | |
| Trust towards the European Parliament 2011 | 4746 | 0.22 | dummy variable | | | |
| Trust towards the police 2011 | 4746 | 0.46 | dummy variable | | | |
| Trust towards the government 2011 | 4746 | 0.18 | dummy variable | | | |
| Trust towards the state social security system 2011 | 4746 | 0.21 | dummy variable | | | |
| Lust for life2011 | 4746 | 8.56 | 9 | 1.73 | 1 | 10 |
| Achieving goals > fun 2011 | 4746 | 0.49 | dummy variable | | | |
| Success depended on her/himself 2011 | 4746 | 0.73 | 1 | .44 | 0 | 1 |
| Belief in democracy 2011 | 4746 | 0.28 | dummy variable | | | |
| Supports Law and Justice 2011 | 4746 | 0.13 | dummy variable | | | |
| Supports no political party 2011 | 4746 | 0.40 | dummy variable | | | |
| Fun is most important 2011 | 4746 | 0.70 | 1 | .46 | 0 | 1 |
| Entire life delightful or pleasing 2011 | 4746 | 0.44 | dummy variable | | | |
| Money - 1 of 3 most important things in life 2011 | 4746 | 0.34 | dummy variable | | | |
| No decrease in energy to work 2011 | 4746 | 0.69 | 1 | .46 | 0 | 1 |
| Reforms in Poland successful 2011 | 4746 | 0.15 | dummy variable | | | |
| Mobile phone 2011 | 4746 | 0.88 | 1 | .33 | 0 | 1 |
| Income 2011 | 4746 | 3645.9 | 3300 | 2299.6 | 100 | 40000 |
| Health benefits 2011 | 4746 | 0.045 | dummy variable | | | |
| No wasching machine 2011 | 4746 | 0.053 | dummy variable | | | |
| No paid TV 2011 | 4746 | 0.13 | dummy variable | | | |
| No independent apartment 2011 | 4746 | 0.031 | dummy variable | | | |
| Size of the living space 2011 | 4746 | 87.8 | 70 | 60.0 | 5 | 861 |
| Social assistance 2011 | 4746 | 0.093 | dummy variable | | | |
| Entrepreneur 2011 | 4746 | 0.028 | dummy variable | | | |
| Self-employed 2011 | 4746 | 0.16 | dummy variable | | | |
| Unemployed 2011 | 4746 | 0.060 | dummy variable | | | |
| Inactive (not retired) 2011 | 4746 | 0.15 | dummy variable | | | |
| Retired (not working) 2011 | 4746 | 0.079 | dummy variable | | | |
| Retired working 2011 | 4746 | 0.017 | dummy variable | | | |
| Inflexible worktime 2011 | 4746 | 0.37 | dummy variable | | | |
| Longer breaks in work impossible 2011 | 4746 | 0.32 | dummy variable | | | |
| Inflexible work (place) 2011 | 4746 | 0.53 | dummy variable | | | |
| Permanent employment 2011 | 4746 | 0.39 | dummy variable | | | |
| Working full time 2011 | 4746 | 0.70 | dummy variable | | | |
| Armed forces 2011 | 4746 | 0.036 | dummy variable | | | |
| Managers and officials 2011 | 4746 | 0.046 | dummy variable | | | |
| Professionals 2011 | 4746 | 0.073 | dummy variable | | | |
| Technicians 2011 | 4746 | 0.069 | dummy variable | | | |
| Clerical support 2011 | 4746 | 0.037 | dummy variable | | | |
| Service and sales 2011 | 4746 | 0.074 | dummy variable | | | |
| Farmers 2011 | 4746 | 0.15 | dummy variable | | | |
| Craft workers 2011 | 4746 | 0.28 | dummy variable | | | |
| Plant/machine operators 2011 | 4746 | 0.15 | dummy variable | | | |
| Elementary occupations 2011 | 4746 | 0.081 | dummy variable | | | |
| Household ratio of adults sport activity 2011 | 4746 | 0.31 | dummy variable | | | |
| Household ratio of u20 sport activity 2011 | 4746 | 0.11 | dummy variable | | | |
| Number of stadiums 2011 | 4746 | 5.73 | 5.29 | 3.27 | 0.71 | 21.3 |
| Number of fields 2011 | 4746 | 2.21 | 2.08 | 1.32 | 0.27 | 7.58 |
| Number of indoor arenas 2011 | 4746 | 1.68 | 1.68 | 0.72 | 0.32 | 3.54 |
| Number of gyms 2011 | 4746 | 1.59 | 1.48 | 0.86 | 0.20 | 5.01 |
| Number of courts 2011 | 4746 | 3.34 | 3.30 | 1.45 | 0.80 | 7.96 |
| Number of golf fields 2011 | 4746 | 2.14 | 0 | 7.18 | 0 | 37.1 |
| Number of swimming pools 2011 | 4746 | 1.44 | 1.32 | 0.78 | 0.31 | 3.86 |
| Number of horse tracks 2011 | 4746 | 1.19 | 0 | 1.91 | 0 | 8.90 |
| Number of shooting 2011 | 4746 | 3.60 | 2.63 | 4.09 | 0 | 25.5 |
| Number of winter 2011 | 4746 | 5.29 | 3.82 | 7.03 | 0 | 51.0 |
| Number of motorsports 2011 | 4746 | 1.56 | 1.46 | 2.03 | 0 | 12.6 |
| Number of outdoor 2011 | 4746 | 4.14 | 3.62 | 2.65 | 0.32 | 12.1 |
| Number of Orliki 2010 | 4746 | 5.02 | 4.82 | 2.25 | 0.48 | 10.8 |

The table presents ratios of ‘1’ values for dummy variables and means, medians, standard deviations, minimum values and maximum values for continuous and categorical variables.

Table C. Balancing variables – descriptive statistics for women.

|  | **count** | **mean** | **median** | **sd** | **min** | **max** |
| --- | --- | --- | --- | --- | --- | --- |
| Household ratio of adults sport activity 2011 | 4027 | 0.31 | dummy variable | | | |
| Household ratio of u20 sport activity 2011 | 4027 | 0.12 | dummy variable | | | |
| Age 2011 | 4027 | 47.2 | 48.00 | 11.1 | 25 | 64 |
| BMI 2011 | 4027 | 25.8 | 25.26 | 4.72 | 15.6 | 54.8 |
| Disability 2011 | 4027 | 0.11 | dummy variable | | | |
| Married 2011 | 4027 | 0.74 | dummy variable | | | |
| Number of children (0-4) 2011 | 4027 | 0.21 | 0.00 | 0.49 | 0 | 3 |
| Number of children (5-9) 2011 | 4027 | 0.22 | 0.00 | 0.49 | 0 | 4 |
| Dissatisfaction with health 2011 | 4027 | 3.02 | 3.00 | 1.19 | 1 | 7 |
| >1 physical activity 2011 | 4027 | 0.091 | dummy variable | | | |
| Physical problems 2011 | 4027 | 0.66 | dummy variable | | | |
| Health problems 2011 | 4027 | 0.60 | dummy variable | | | |
| Seriously ill 2010 | 4027 | 0.12 | dummy variable | | | |
| Tiredness (<1/2) 2011 | 4027 | 0.38 | dummy variable | | | |
| Tiredness (>1/2) 2011 | 4027 | 0.075 | dummy variable | | | |
| Too much alcohol 2010 | 4027 | 0.019 | dummy variable | | | |
| No decrease in energy to work 2011 | 4027 | 0.67 | dummy variable | | | |
| Entire life delightful or pleasing 2011 | 4027 | 0.42 | dummy variable | | | |
| Lust for life2011 | 4027 | 8.59 | 9.00 | 1.74 | 1 | 10 |
| Achieving goals > fun 2011 | 4027 | 0.63 | dummy variable | | | |
| Success depended on her/himself 2011 | 4027 | 0.68 | dummy variable | | | |
| Fun is most important 2011 | 4027 | 0.61 | dummy variable | | | |
| Number of friends met 2011 | 4027 | 5.03 | 4.00 | 4.99 | 0 | 60 |
| Number of acquaintances met 2011 | 4027 | 6.24 | 4.00 | 7.21 | 0 | 70 |
| Number of friends 2011 | 4027 | 6.51 | 5.00 | 6.52 | 0 | 99 |
| General trust 2011 | 4027 | 0.14 | dummy variable | | | |
| Work for local society 2011 | 4027 | 0.17 | dummy variable | | | |
| Member of organisations 2011 | 4027 | 0.20 | 0.00 | 0.49 | 0 | 3 |
| Public meetings 2011 | 4027 | 0.27 | dummy variable | | | |
| Voluntary activities 2011 | 4027 | 0.19 | dummy variable | | | |
| Voted in 2010 | 4027 | 0.75 | dummy variable | | | |

The table presents ratios of ‘1’ values for dummy variables and means, medians, standard deviations, minimum values and maximum values for continuous and categorical variables.

Table D. Balancing variables – descriptive statistics for men.

|  | **count** | **mean** | **median** | **sd** | **min** | **max** |
| --- | --- | --- | --- | --- | --- | --- |
| Household ratio of adults sport activity 2011 | 3034 | 0.31 | dummy variable | | | |
| Household ratio of u20 sport activity 2011 | 3034 | 0.11 | dummy variable | | | |
| Age 2011 | 3034 | 46.6 | dummy variable | | | |
| BMI 2011 | 3034 | 27.3 | 26.83 | 4.03 | 16.3 | 62.1 |
| Disability 2011 | 3034 | 0.14 | dummy variable | | | |
| Married 2011 | 3034 | 0.77 | dummy variable | | | |
| Number of children (0-4) 2011 | 3034 | 0.23 | .00 | .50 | 0 | 3 |
| Number of children (5-9) 2011 | 3034 | 0.21 | .00 | .49 | 0 | 4 |
| Dissatisfaction with health 2011 | 3034 | 2.86 | 3.00 | 1.20 | 1 | 7 |
| >1 physical activity 2011 | 3034 | 0.10 | dummy variable | | | |
| Physical problems 2011 | 3034 | 0.62 | dummy variable | | | |
| Health problems 2011 | 3034 | 0.55 | dummy variable | | | |
| Seriously ill 2010 | 3034 | 0.10 | dummy variable | | | |
| Tiredness (<1/2) 2011 | 3034 | 0.31 | dummy variable | | | |
| Tiredness (>1/2) 2011 | 3034 | 0.047 | dummy variable | | | |
| Too much alcohol 2010 | 3034 | 0.13 | dummy variable | | | |
| No decrease in energy to work 2011 | 3034 | 0.70 | dummy variable | | | |
| Entire life delightful or pleasing 2011 | 3034 | 0.45 | dummy variable | | | |
| Lust for life2011 | 3034 | 8.56 | 9.00 | 1.70 | 1 | 10 |
| Achieving goals > fun 2011 | 3034 | 0.50 | dummy variable | | | |
| Success depended on her/himself 2011 | 3034 | 0.74 | dummy variable | | | |
| Fun is most important 2011 | 3034 | 0.70 | dummy variable | | | |
| Number of friends met 2011 | 3034 | 5.54 | 4.00 | 5.64 | 0 | 60 |
| Number of acquaintances met 2011 | 3034 | 6.87 | 4.00 | 7.99 | 0 | 80 |
| Number of friends 2011 | 3034 | 7.09 | 5.00 | 7.84 | 0 | 99 |
| General trust 2011 | 3034 | 0.15 | dummy variable | | | |
| Work for local society 2011 | 3034 | 0.20 | dummy variable | | | |
| Member of organisations 2011 | 3034 | 0.22 | 0.00 | .55 | 0 | 3 |
| Public meetings 2011 | 3034 | 0.31 | dummy variable | | | |
| Voluntary activities 2011 | 3034 | 0.26 | dummy variable | | | |
| Voted in 2010 | 3034 | 0.72 | dummy variable | | | |

The table presents ratios of ‘1’ values for dummy variables and means, medians, standard deviations, minimum values and maximum values for continuous and categorical variables.

Table E. Outcome variables – descriptive statistics for women.

|  | **count** | **mean** | **median** | **sd** | **min** | **max** |
| --- | --- | --- | --- | --- | --- | --- |
| Sport activity (at least one type of activity) 2015 | 4014 | 0.34 | dummy variable | | | |
| >1 physical activity 2015 | 4027 | 0.10 | dummy variable | | | |
| BMI 2015 | 4013 | 26.4 | 25.8 | 4.76 | 14.8 | 54.7 |
| Dissatisfaction with health 2015 | 4019 | 3.08 | 3 | 1.18 | 1 | 7 |
| Physical problems 2015 | 4027 | 0.69 | dummy variable | | | |
| Health problems 2015 | 4027 | 0.62 | dummy variable | | | |
| Seriously ill 2014 | 4027 | 0.12 | dummy variable | | | |
| Tiredness not-related to work 2015 | 4027 | 0.47 | dummy variable | | | |
| Too much alcohol 2014 | 4027 | 0.023 | dummy variable | | | |
| No decrease in energy to work 2015 | 4027 | 0.62 | dummy variable | | | |
| Entire life delightful or pleasing 2015 | 4027 | 0.42 | dummy variable | | | |
| Lust for life2015 | 4023 | 8.64 | 9 | 1.67 | 1 | 10 |
| Achieving goals > fun 2015 | 4027 | 0.65 | dummy variable | | | |
| Success depended on her/himself 2015 | 4027 | 0.70 | dummy variable | | | |
| Fun is most important 2015 | 4027 | 0.57 | dummy variable | | | |
| Member of sports club 2015 | 4027 | .0027 | dummy variable | | | |
| Number of friends 2015 | 4015 | 5.97 | 4 | 5.83 | 0 | 60 |
| Number of acquaintances met 2015 | 3990 | 5.93 | 4 | 6.89 | 0 | 90 |
| Number of friends met 2015 | 4009 | 4.72 | 4 | 4.72 | 0 | 90 |
| General trust 2015 | 4017 | 0.16 | dummy variable | | | |
| Work for local society 2015 | 4027 | 0.16 | dummy variable | | | |
| Member of organisations 2015 | 4027 | 0.18 | 0 | 0.49 | 0 | 3 |
| Public meetings 2015 | 4027 | 0.22 | dummy variable | | | |
| Voluntary activities 2015 | 4027 | 0.075 | dummy variable | | | |
| Voted in 2014 | 4027 | 0.75 | dummy variable | | | |

The table presents ratios of ‘1’ values for dummy variables and means, medians, standard deviations, minimum values and maximum values for continuous and categorical variables.

Table F. Outcome variables – descriptive statistics for men.

|  | **count** | **mean** | **median** | **sd** | **min** | **max** |
| --- | --- | --- | --- | --- | --- | --- |
| Sport activity (at least one type of activity) 2015 | 3028 | 0.32 | dummy variable | | | |
| >1 physical activity 2015 | 3034 | 0.11 | dummy variable | | | |
| BMI 2015 | 3029 | 27.6 | 27.2 | 4.04 | 14.2 | 51.7 |
| Dissatisfaction with health 2015 | 3024 | 2.96 | 3 | 1.20 | 1 | 7 |
| Physical problems 2015 | 3034 | 0.66 | dummy variable | | | |
| Health problems 2015 | 3034 | 0.57 | dummy variable | | | |
| Seriously ill 2014 | 3034 | 0.12 | dummy variable | | | |
| Tiredness not-related to work 2015 | 3034 | 0.37 | dummy variable | | | |
| Too much alcohol 2014 | 3034 | 0.11 | dummy variable | | | |
| No decrease in energy to work 2015 | 3034 | 0.65 | dummy variable | | | |
| Entire life delightful or pleasing 2015 | 3034 | 0.44 | dummy variable | | | |
| Lust for life2015 | 3032 | 8.56 | 9 | 1.68 | 1 | 10 |
| Achieving goals > fun 2015 | 3034 | 0.57 | dummy variable | | | |
| Success depended on her/himself 2015 | 3034 | 0.75 | dummy variable | | | |
| Fun is most important 2015 | 3034 | 0.65 | dummy variable | | | |
| Member of sports club 2015 | 3034 | 0.016 | dummy variable | | | |
| Number of friends 2015 | 3025 | 6.31 | 5 | 6.77 | 0 | 60 |
| Number of acquaintances met 2015 | 3018 | 6.45 | 4 | 8.11 | 0 | 99 |
| Number of friends met 2015 | 3022 | 4.96 | 4 | 4.78 | 0 | 50 |
| General trust 2015 | 3025 | 0.15 | dummy variable | | | |
| Work for local society 2015 | 3034 | 0.18 | dummy variable | | | |
| Member of organisations 2015 | 3034 | 0.19 | 0 | 0.52 | 0 | 3 |
| Public meetings 2015 | 3034 | 0.25 | dummy variable | | | |
| Voluntary activities 2015 | 3034 | 0.077 | dummy variable | | | |
| Voted in 2014 | 3034 | 0.74 | dummy variable | | | |

The table presents ratios of ‘1’ values for dummy variables and means, medians, standard deviations, minimum values and maximum values for continuous and categorical variables.
