# Supplementary material for: Impact of sports activity on Polish adults: Self-reported health, social capital & attitudes
Source: PLoS One. 2019 Dec 19;14(12):e0226812. doi: 10.1371/journal.pone.0226812 (PMC6922371; doi:10.1371/journal.pone.0226812)
Supplement: S3 Appendix — (DOCX) [file pone.0226812.s003.docx]

# S3 Appendix. Results of the probit model estimations.

Table A. Results of probit model estimaton for women – stratification based on age.

| **Dependent variable: Sport activity (at least one type of activity) 2013** | **Age 41-65** | | **Age 25-40** | |
| --- | --- | --- | --- | --- |
|  | **non-active women** | **active women** | **non-active women** | **active women** |
| Large city (>500k) | -0.729*** | -0.459 | 0.204 | 0.623 |
| Big city (200-500k) | -0.128 | 0.214 | 0.059 | 0.375 |
| Medium city (100-200k) | -0.103 | 0.147 | -0.036 | 0.706** |
| Small city (20-100k) | -0.125 | 0.213 | -0.115 | 0.344* |
| Town (<20k) | -0.194** | -0.222 | -0.248 | 0.528** |
| kujawsko-pomorskie | 0.032 | -0.082 | -0.339 | -0.126 |
| lubelskie | -0.113 | -0.387 | 0.006 | -0.362 |
| lubuskie | -0.254 | -0.161 | -0.252 | 0.124 |
| łódzkie | -0.339 | -0.240 | -0.076 | -0.013 |
| małopolskie | -0.726*** | -0.865** | -0.538 | 0.206 |
| mazowieckie | -0.567** | -0.559 | 0.138 | 0.598 |
| opolskie | 0.004 | -0.201 | 0.255 | -0.039 |
| podkarpackie | -0.163 | -0.878* | -0.661 | 0.055 |
| podlaskie | -0.662** | 0.008 | 0.110 | -0.200 |
| pomorskie | -0.230 | -0.551 | 0.074 | 1.109** |
| śląskie | -0.536** | -0.965*** | 0.249 | 0.988** |
| świętokrzyskie | -0.858*** | -0.451 | 0.647 | -0.306 |
| warmińsko-mazurskie | -0.405 | 0.258 | -0.712 | -0.291 |
| wielkopolskie | -0.256 | -0.032 | 0.102 | -0.028 |
| zachodniopomorskie | -0.408 | 0.347 | -0.221 | 0.122 |
| Married 2011 | -0.092 | -0.565 | 0.121 | 1.079*** |
| Married 2013 | -0.075 | 0.359 | -0.350* | -0.711** |
| Number of children (0-4) 2011 | -0.107 | 0.059 | -0.121 | -0.085 |
| Number of children (5-9) 2011 | -0.055 | -0.234 | 0.103 | 0.064 |
| Number of teens (10-14) 2011 | 0.013 | -0.212** | 0.076 | 0.096 |
| Number of teens (15-19) 2011 | -0.075 | -0.210** | -0.043 | 0.582*** |
| Number of adults 2011 | -0.011 | -0.038 | 0.001 | -0.084 |
| Number of children (0-1) 2013 | 0.116 | -0.048 | -0.497** | -0.790*** |
| Higher education, father 2011 | 0.355** | 0.111 | -0.213 | -0.126 |
| Educated her/himself 2011 | -0.002 | -0.084 | 0.335** | -0.092 |
| Formal education 2011 | -0.498 | 0.576 | -0.120 | 0.504* |
| Too little time with the child 2011 | -0.001 | 0.078 | -0.287 | 0.171 |
| Overburdened with work duties 2011 | 0.149 | -0.294 | -0.008 | -0.559* |
| Number of social meetings 2011 | 0.002 | 0.045** | 0.033 | 0.066** |
| Number of entertainment 2011 | 0.105* | 0.152** | 0.047 | 0.167** |
| Number of restaurants 2011 | 0.020 | 0.008 | 0.110*** | -0.064* |
| Time using computer 2011 | 0.004 | 0.000 | 0.002 | 0.003 |
| Number of cigarettes 2011 | -0.009** | -0.009 | -0.030*** | -0.026** |
| Too much alcohol 2010 | -0.375 | -0.019 | -0.181 | 2.040*** |
| Drugs/designer drugs 2011 | 0.101 | - | 0.702 | - |
| New skills 2011 | 0.024 | -0.095 | -0.019 | 0.068 |
| Age 2011 | -0.006 | -0.008 | -0.019 | -0.068*** |
| BMI 2011 | 0.004 | 0.002 | 0.011 | 0.035* |
| Dissatisfaction with health 2011 | -0.041 | -0.021 | 0.032 | -0.142* |
| Physical problems 2011 | 0.013 | -0.207 | -0.034 | 0.089 |
| Health problems 2011 | -0.075 | -0.014 | -0.018 | -0.079 |
| Rare suicidal thoughts 2011 | -0.194* | -0.063 | 0.135 | -0.130 |
| Often suicidal thoughts 2011 | -0.179 | -0.167 | 0.351 | -0.106 |
| Seriously ill 2010 | 0.078 | 0.114 | 0.100 | -0.230 |
| Disability 2011 | -0.009 | -0.520*** | -0.020 | 0.179 |
| Problems with sleeping 2011 | -0.081 | -0.107 | 0.014 | -0.107 |
| Some worries about digestive system 2011 | -0.096 | -0.336*** | -0.087 | 0.180 |
| Constant worries about health 2011 | 0.014 | -0.291* | -0.418* | 0.382 |
| Losing interest in sex 2011 | -0.005 | 0.260** | -0.133 | -0.096 |
| Headaches (<1/2) 2011 | -0.073 | -0.075 | 0.088 | 0.005 |
| Headaches (>1/2) 2011 | 0.158 | -0.195 | 0.143 | 0.808*** |
| Stomach pains (<1/2) 2011 | -0.109 | -0.005 | 0.052 | 0.083 |
| Stomach pains (>1/2) 2011 | 0.032 | 0.209 | 0.784*** | -0.544 |
| Pain in neck or arm muscles (<1/2) 2011 | 0.053 | 0.017 | 0.060 | 0.075 |
| Pain in neck or arm muscles (>1/2) 2011 | -0.004 | 0.226 | 0.114 | -0.056 |
| Chest or heart pains (<1/2) 2011 | 0.031 | -0.081 | -0.166 | -0.133 |
| Chest or heart pains (>1/2) 2011 | 0.233 | 0.282 | 0.326 | -0.743 |
| Dry mouth or throat (<1/2) 2011 | -0.055 | -0.071 | 0.117 | 0.315* |
| Dry mouth or throat (>1/2) 2011 | -0.059 | -0.094 | 0.591** | 0.862** |
| Sweating (<1/2) 2011 | -0.035 | 0.106 | 0.101 | 0.087 |
| Sweating (>1/2) 2011 | 0.140 | 0.249 | -0.295 | -0.320 |
| Shortness of breath (<1/2) 2011 | 0.143* | -0.201* | 0.201 | 0.268 |
| Shortness of breath (>1/2) 2011 | 0.048 | 0.656** | 0.554 | 0.484 |
| Body pains (<1/2) 2011 | -0.009 | -0.022 | 0.291** | -0.306** |
| Body pains (>1/2) 2011 | -0.186 | 0.250 | 0.268 | -0.689 |
| Palpitation (<1/2) 2011 | -0.090 | -0.052 | -0.182 | -0.096 |
| Palpitation (>1/2) 2011 | 0.059 | -0.504* | 0.201 | 0.605 |
| Shivers or convulsions (<1/2) 2011 | 0.142 | -0.125 | -0.635*** | 0.087 |
| Shivers or convulsions (>1/2) 2011 | 0.179 | -0.440 | -0.689 | -1.172* |
| Pressure on bladder (<1/2) 2011 | -0.040 | 0.310*** | -0.176 | -0.033 |
| Pressure on bladder (>1/2) 2011 | -0.173 | 0.467** | -0.306 | -0.310 |
| Tiredness (<1/2) 2011 | 0.140** | 0.000 | -0.037 | -0.138 |
| Tiredness (>1/2) 2011 | 0.088 | -0.186 | -0.126 | -0.030 |
| Constipation (<1/2) 2011 | 0.242*** | -0.028 | 0.149 | -0.172 |
| Constipation (>1/2) 2011 | 0.095 | -0.397* | 0.205 | 0.667* |
| Nosebleeds (<1/2) 2011 | -0.128 | 0.369* | 0.048 | -0.284 |
| Nosebleeds (>1/2) 2011 | 0.067 | -0.022 | -0.916 | 1.646* |
| Blood pressure (<1/2) 2011 | 0.139* | -0.188* | 0.221 | 0.488** |
| Blood pressure (>1/2) 2011 | 0.102 | 0.051 | -0.173 | -0.110 |
| Years of education 2011 | 0.040** | 0.019 | -0.034 | -0.029 |
| Higher Education 2011 | -0.105 | -0.006 | 0.287 | -0.070 |
| English 2011 | 0.109 | -0.270 | 0.143 | 0.066 |
| German 2011 | -0.233 | -0.153 | 0.011 | 0.129 |
| French 2011 | 0.261 | 0.240 | 0.309 | -0.333 |
| Russian 2011 | 0.017 | -0.134 | 0.092 | 0.683*** |
| Spanish 2011 | - | -0.144 | -1.187 | -0.743 |
| other language 2011 | -0.376 | 0.117 | - | -0.277 |
| No need for books 2011 | 0.006 | 0.017 | 0.058 | 0.229 |
| No books 2011 | -0.220* | -0.020 | 0.159 | 0.127 |
| Afraid of neighbourhood 2011 | 0.140 | -0.146 | 0.083 | 0.217 |
| Voted in 2014 2010 | 0.083 | -0.151 | 0.113 | 0.165 |
| Number of social meetings 2011 | 0.033*** | 0.040** | 0.044** | 0.011 |
| Public meetings 2011 | 0.104 | 0.049 | -0.054 | 0.125 |
| Member of organisations 2011 | -0.098 | -0.168* | -0.124 | 0.016 |
| Fullfiling roles in organisations 2011 | -0.029 | 0.388* | -0.083 | 0.055 |
| Voluntary activities 2011 | 0.063 | -0.014 | 0.070 | -0.118 |
| Number of friends met 2011 | 0.007 | 0.045*** | 0.008 | 0.036** |
| Number of acquaintances met 2011 | -0.003 | 0.009 | -0.006 | 0.001 |
| Number of family members met 2011 | 0.001 | 0.000 | -0.001 | -0.008 |
| Number of friends 2011 | 0.012** | -0.013* | 0.001 | -0.018 |
| Work for local society 2011 | 0.070 | 0.237** | 0.219 | 0.140 |
| General trust 2011 | 0.022 | -0.033 | 0.044 | 0.293 |
| Trust towards banks 2011 | 0.061 | -0.096 | -0.124 | 0.143 |
| Trust towards Parliament 2011 | -0.178 | -0.155 | -0.260 | -0.001 |
| Trust towards the president 2011 | 0.007 | -0.096 | 0.004 | 0.409** |
| Trust towards stock exchange 2011 | 0.111 | -0.514** | -0.286 | 0.278 |
| Trust towards the NBP 2011 | 0.223*** | 0.014 | 0.041 | -0.103 |
| Trust towards the family 2011 | -0.074 | 0.020 | -0.100 | -0.217 |
| Trust towards the neighbours 2011 | -0.089 | -0.089 | 0.049 | -0.020 |
| Trust towards nonstate pension funds 2011 | -0.204* | 0.029 | 0.134 | 0.465** |
| Trust towards courts 2011 | -0.069 | -0.048 | -0.173 | 0.050 |
| Trust towards the European Parliament 2011 | 0.129 | 0.187 | 0.109 | 0.139 |
| Trust towards the police 2011 | -0.141** | -0.074 | -0.064 | -0.060 |
| Trust towards the government 2011 | 0.146 | 0.374*** | 0.340* | -0.680*** |
| Trust towards the state social security system 2011 | -0.141* | -0.117 | -0.011 | -0.231 |
| Lust for life2011 | -0.011 | -0.043 | 0.046 | 0.041 |
| Achieving goals > fun 2011 | -0.095 | -0.219** | -0.128 | -0.009 |
| Success depended on her/himself 2011 | -0.003 | -0.084 | 0.022 | -0.115 |
| Belief in democracy 2011 | -0.038 | -0.039 | 0.093 | 0.310** |
| Supports Law and Justice 2011 | -0.061 | 0.258* | 0.310* | -0.152 |
| Supports no political party 2011 | 0.100 | -0.007 | 0.078 | 0.107 |
| Fun is most important 2011 | 0.093 | 0.075 | -0.034 | -0.328** |
| Entire life delightful or pleasing 2011 | 0.058 | -0.011 | 0.047 | -0.182 |
| Money - 1 of 3 most important things in life 2011 | -0.100 | -0.182 | 0.026 | -0.017 |
| No decrease in energy to work 2011 | 0.085 | 0.039 | 0.411*** | 0.121 |
| Reforms in Poland successful 2011 | 0.010 | 0.084 | -0.175 | -0.077 |
| Mobile phone 2011 | 0.123 | 0.289** | 0.659** | 0.162 |
| Income 2011 | 0.000 | 0.000 | 0.000 | 0.000 |
| Health benefits 2011 | 0.099 | 0.669*** | 0.229 | -0.260 |
| No wasching machine 2011 | 0.073 | 0.156 | 0.174 | 0.769* |
| No paid TV 2011 | -0.020 | -0.116 | -0.061 | -0.445** |
| No independent apartment 2011 | -0.006 | 0.027 | -0.314 | 0.400 |
| Size of the living space 2011 | 0.001 | 0.000 | 0.000 | 0.003* |
| Social assistance 2011 | 0.120 | -0.077 | -0.235* | -0.749*** |
| Entrepreneur 2011 | -0.012 | -0.373 | 0.287 | -0.156 |
| Self-employed 2011 | -0.083 | -0.472** | -0.072 | -0.187 |
| Unemployed 2011 | 0.252* | -0.134 | 0.123 | -0.115 |
| Inactive (not retired) 2011 | -0.146 | -0.133 | -0.028 | -0.230 |
| Retired (not working) 2011 | -0.008 | -0.334* | - | - |
| Retired working 2011 | -0.134 | 0.757** | - | - |
| Inflexible worktime 2011 | -0.069 | -0.026 | 0.035 | -0.065 |
| Longer breaks in work impossible 2011 | 0.195* | -0.020 | -0.113 | 0.347** |
| Inflexible work (place) 2011 | -0.090 | -0.311** | -0.049 | -0.378** |
| Permanent employment 2011 | 0.075 | 0.108 | -0.170 | 0.215 |
| Working full time 2011 | -0.032 | -0.071 | 0.127 | 0.021 |
| Managers and officials 2011 | 0.133 | 0.653** | -0.074 | 0.110 |
| Professionals 2011 | -0.061 | 0.603** | 0.369 | 0.132 |
| Technicians 2011 | -0.162 | 0.662** | 0.605** | 0.188 |
| Clerical support 2011 | 0.057 | 0.530* | 0.626*** | -0.125 |
| Service and sales 2011 | 0.131 | 0.140 | 0.152 | -0.479* |
| Farmers 2011 | 0.024 | 0.778*** | -0.104 | 0.069 |
| Craft workers 2011 | 0.006 | 0.326 | 0.665*** | -0.541 |
| Plant/machine operators 2011 | -0.153 | 0.527 | -0.405 | 0.532 |
| Elementary occupations 2011 | 0.116 | 0.601** | 0.190 | -0.358 |
| Household ratio of adults sport activity 2011 | 0.179** | 0.493*** | 0.021 | -0.050 |
| Household ratio of u20 sport activity 2011 | -0.008 | -0.116 | 0.004 | -0.363 |
| Number of stadiums 2011 | -0.032 | 0.021 | 0.045 | 0.023 |
| Number of fields 2011 | -0.044 | 0.031 | -0.004 | 0.052 |
| Number of indoor arenas 2011 | 0.027 | 0.108 | -0.041 | -0.123 |
| Number of gyms 2011 | -0.065 | 0.037 | 0.206** | 0.150 |
| Number of courts 2011 | 0.032 | 0.033 | 0.050 | -0.108 |
| Number of golf fields 2011 | -0.010* | -0.013 | 0.010 | -0.001 |
| Number of swimming pools 2011 | 0.048 | -0.076 | -0.247** | -0.077 |
| Number of horse tracks 2011 | 0.015 | -0.014 | 0.054 | -0.047 |
| Number of shooting 2011 | -0.002 | -0.026 | 0.000 | 0.059** |
| Number of winter 2011 | -0.007 | -0.005 | 0.002 | 0.010 |
| Number of motorsports 2011 | 0.012 | -0.015 | -0.013 | 0.030 |
| Number of outdoor 2011 | -0.006 | 0.007 | -0.016 | -0.094** |
| Number of Orliki 2010 | -0.004 | -0.160*** | 0.015 | 0.094 |
| Constant | -.572 | 1.259 | -2.083** | 1.003 |
| Observations | 2,954 | 1,204 | 1,216 | 741 |
| Pseudo R-squared | 0.123 | 0.210 | 0.202 | 0.279 |

The table presents results of first-step probit estimation for women stratified by age and past SA. For each stratum, coefficients are reported along with significance, denoted using asterisks: *** p < 0.010, ** p < 0.050, * p < 0.100.

Table B. Results of probit model estimaton for men – stratification based on age.

| **Dependent variable: Sport activity (at least one type of activity) 2013** | **Age 41-65** | | **Age 25-40** | |
| --- | --- | --- | --- | --- |
|  | **non-active men** | **active men** | **non-active men** | **active men** |
| Large city (>500k) | -0.365 | 0.625* | 0.361 | 0.649 |
| Big city (200-500k) | -0.395** | 0.732*** | -0.039 | 0.358 |
| Medium city (100-200k) | -0.078 | 0.280 | -0.348 | -0.089 |
| Small city (20-100k) | 0.076 | 0.657*** | 0.180 | 0.324 |
| Town (<20k) | 0.126 | 0.473*** | 0.311* | -0.036 |
| kujawsko-pomorskie | -0.186 | -0.751 | -0.163 | 0.460 |
| lubelskie | -0.285 | -0.545 | 0.465 | 0.043 |
| lubuskie | -0.840** | -0.038 | 0.334 | -0.561 |
| łódzkie | -0.313 | -0.827* | -0.424 | -0.168 |
| małopolskie | -0.322 | -0.607 | -0.104 | -0.445 |
| mazowieckie | -0.835** | -0.322 | -0.190 | -0.466 |
| opolskie | 0.409 | -0.239 | 0.200 | 0.049 |
| podkarpackie | 0.064 | -0.001 | -0.475 | 0.074 |
| podlaskie | -0.734** | -0.453 | -0.115 | -0.670 |
| pomorskie | -0.281 | -0.612 | -0.421 | -0.248 |
| śląskie | -0.225 | -0.905** | -0.217 | 0.196 |
| świętokrzyskie | -0.253 | -1.114** | 0.697 | -1.257** |
| warmińsko-mazurskie | -1.034*** | 0.553 | -0.305 | -0.522 |
| wielkopolskie | -0.170 | -0.658 | 0.238 | -0.647 |
| zachodniopomorskie | -0.266 | -1.094** | 0.526 | 0.006 |
| Married 2011 | -0.410 | 0.147 | -0.392 | 0.241 |
| Married 2013 | 0.408 | -0.486 | 0.204 | -0.530 |
| Number of children (0-4) 2011 | -0.185* | 0.085 | -0.118 | -0.243* |
| Number of children (5-9) 2011 | 0.037 | -0.151 | -0.032 | -0.149 |
| Number of teens (10-14) 2011 | 0.074 | -0.065 | -0.037 | -0.325** |
| Number of teens (15-19) 2011 | -0.133* | 0.067 | -0.118 | 0.239 |
| Number of adults 2011 | -0.047 | -0.104** | -0.003 | -0.046 |
| Number of children (0-1) 2013 | 0.259 | 0.483 | -0.589*** | -0.345 |
| Higher education, father 2011 | 0.186 | -0.557** | 0.626** | 0.038 |
| Educated her/himself 2011 | -0.428** | -0.059 | -0.387* | -0.146 |
| Formal education 2011 | -0.647 | - | 0.046 | 0.504 |
| Too little time with the child 2011 | 0.095 | -0.481** | -0.353* | 0.300 |
| Overburdened with work duties 2011 | 0.085 | 0.659*** | 0.036 | 0.396 |
| Number of social meetings 2011 | 0.017 | 0.011 | 0.009 | -0.041* |
| Number of entertainment 2011 | -0.096 | -0.118 | -0.109 | 0.141** |
| Number of restaurants 2011 | 0.013 | -0.001 | 0.072*** | -0.011 |
| Time using computer 2011 | 0.012*** | -0.007 | -0.006 | -0.010** |
| Number of cigarettes 2011 | -0.009** | -0.013** | -0.007 | -0.004 |
| Too much alcohol 2010 | -0.014 | 0.099 | -0.073 | 0.119 |
| Drugs/designer drugs 2011 | - | - | -0.013 | 0.065 |
| New skills 2011 | 0.181 | 0.178 | -0.306* | -0.015 |
| Age 2011 | 0.017** | -0.005 | 0.002 | -0.026 |
| BMI 2011 | 0.004 | -0.008 | -0.004 | 0.033* |
| Dissatisfaction with health 2011 | -0.073* | 0.006 | 0.081 | 0.042 |
| Physical problems 2011 | 0.152 | -0.099 | -0.034 | 0.297 |
| Health problems 2011 | -0.093 | 0.245* | -0.057 | -0.231 |
| Rare suicidal thoughts 2011 | 0.096 | 0.465** | -0.001 | -0.050 |
| Often suicidal thoughts 2011 | 0.039 | -0.205 | -0.125 | -0.433 |
| Seriously ill 2010 | 0.173 | -0.037 | 0.239 | -0.710** |
| Disability 2011 | -0.146 | -0.168 | -0.297 | 0.076 |
| Problems with sleeping 2011 | 0.071 | -0.026 | 0.239 | 0.876** |
| Some worries about digestive system 2011 | 0.048 | 0.143 | -0.490*** | -0.223 |
| Constant worries about health 2011 | 0.194 | 0.197 | -0.377 | -1.130* |
| Losing interest in sex 2011 | -0.204** | -0.064 | 0.020 | 0.462** |
| Headaches (<1/2) 2011 | 0.017 | -0.237* | -0.040 | 0.073 |
| Headaches (>1/2) 2011 | -0.191 | -0.012 | 0.230 | 0.136 |
| Stomach pains (<1/2) 2011 | -0.007 | 0.031 | -0.020 | 0.063 |
| Stomach pains (>1/2) 2011 | 0.153 | -0.139 | -0.655 | 0.112 |
| Pain in neck or arm muscles (<1/2) 2011 | 0.049 | -0.181 | 0.078 | -0.027 |
| Pain in neck or arm muscles (>1/2) 2011 | 0.184 | -0.109 | -0.444 | 0.082 |
| Chest or heart pains (<1/2) 2011 | -0.033 | -0.427*** | 0.120 | -0.245 |
| Chest or heart pains (>1/2) 2011 | -0.324 | -0.620** | 0.531 | -1.447* |
| Dry mouth or throat (<1/2) 2011 | -0.094 | 0.132 | -0.266 | -0.052 |
| Dry mouth or throat (>1/2) 2011 | 0.145 | -0.380 | 0.391 | 1.140* |
| Sweating (<1/2) 2011 | -0.056 | 0.056 | 0.475** | 0.466* |
| Sweating (>1/2) 2011 | -0.285 | 0.136 | 0.798** | -0.140 |
| Shortness of breath (<1/2) 2011 | -0.003 | -0.223 | 0.048 | -0.027 |
| Shortness of breath (>1/2) 2011 | 0.152 | -0.445 | 0.792 | -0.686 |
| Body pains (<1/2) 2011 | -0.059 | -0.065 | 0.007 | -0.299 |
| Body pains (>1/2) 2011 | -0.199 | -0.096 | 0.388 | -0.687 |
| Palpitation (<1/2) 2011 | -0.029 | 0.401** | -0.943*** | 0.512 |
| Palpitation (>1/2) 2011 | 0.201 | 0.741* | -0.618 | - |
| Shivers or convulsions (<1/2) 2011 | -0.081 | -0.262 | -0.038 | 0.085 |
| Shivers or convulsions (>1/2) 2011 | 0.613* | 1.945*** | -0.715 | - |
| Pressure on bladder (<1/2) 2011 | 0.106 | 0.012 | 0.075 | -0.326 |
| Pressure on bladder (>1/2) 2011 | 0.388** | 0.036 | 0.088 | -0.600 |
| Tiredness (<1/2) 2011 | 0.105 | 0.246** | -0.059 | 0.133 |
| Tiredness (>1/2) 2011 | -0.401** | 0.609* | -0.655** | 0.875* |
| Constipation (<1/2) 2011 | 0.178 | -0.005 | 0.453** | 0.621* |
| Constipation (>1/2) 2011 | 0.182 | -0.253 | - | 0.158 |
| Nosebleeds (<1/2) 2011 | 0.057 | -0.002 | -0.082 | 0.156 |
| Nosebleeds (>1/2) 2011 | 0.020 | -0.184 | 1.353* | - |
| Blood pressure (<1/2) 2011 | -0.088 | -0.217 | -0.080 | 0.229 |
| Blood pressure (>1/2) 2011 | -0.049 | -0.310 | -1.166* | 0.041 |
| Years of education 2011 | 0.002 | 0.080** | 0.076** | 0.133*** |
| Higher Education 2011 | 0.203 | -0.104 | -0.195 | -0.607** |
| English 2011 | -0.034 | 0.595*** | 0.005 | 0.595*** |
| German 2011 | -0.037 | -0.420* | -0.272 | -0.509** |
| French 2011 | 0.154 | -0.383 | 0.449 | 1.166** |
| Russian 2011 | 0.031 | 0.010 | -0.013 | -0.347 |
| Spanish 2011 | 1.577** | - | -1.144 | -1.142* |
| other language 2011 | -0.149 | 0.543 | 0.450 | - |
| No need for books 2011 | 0.015 | 0.004 | 0.128 | -0.646*** |
| No books 2011 | -0.326** | -0.835*** | 0.060 | 0.150 |
| Afraid of neighbourhood 2011 | -0.302 | 0.754*** | 0.313 | -0.611 |
| Voted in 2014 2010 | 0.146* | 0.104 | 0.152 | 0.388** |
| Number of social meetings 2011 | -0.020 | -0.001 | 0.011 | -0.005 |
| Public meetings 2011 | 0.089 | -0.149 | 0.226 | 0.217 |
| Member of organisations 2011 | -0.077 | 0.304*** | 0.234 | 0.493*** |
| Fullfiling roles in organisations 2011 | 0.282 | -0.219 | -0.571 | 0.219 |
| Voluntary activities 2011 | -0.001 | 0.242* | 0.078 | 0.059 |
| Number of friends met 2011 | 0.001 | 0.021* | -0.024* | 0.027* |
| Number of acquaintances met 2011 | 0.003 | 0.007 | 0.010 | -0.003 |
| Number of family members met 2011 | 0.005 | 0.005 | 0.019** | -0.031*** |
| Number of friends 2011 | 0.005 | 0.007 | -0.007 | -0.021 |
| Work for local society 2011 | -0.016 | -0.237 | 0.199 | -0.029 |
| General trust 2011 | -0.056 | 0.156 | 0.232 | 0.143 |
| Trust towards banks 2011 | 0.090 | -0.278* | 0.164 | -0.052 |
| Trust towards Parliament 2011 | 0.070 | -0.176 | 0.242 | -0.488** |
| Trust towards the president 2011 | -0.130 | -0.075 | 0.016 | -0.010 |
| Trust towards stock exchange 2011 | -0.317* | -0.256 | 0.061 | -0.471** |
| Trust towards the NBP 2011 | 0.134 | 0.309** | 0.311** | -0.089 |
| Trust towards the family 2011 | -0.226* | -0.276 | -0.189 | 0.730*** |
| Trust towards the neighbours 2011 | -0.213*** | -0.042 | -0.022 | -0.420*** |
| Trust towards nonstate pension funds 2011 | 0.020 | -0.380** | -0.105 | 0.551** |
| Trust towards courts 2011 | -0.049 | 0.200 | -0.213 | 0.031 |
| Trust towards the European Parliament 2011 | 0.068 | 0.297** | -0.118 | 0.731*** |
| Trust towards the police 2011 | 0.259*** | -0.008 | 0.020 | 0.288* |
| Trust towards the government 2011 | -0.029 | -0.275 | -0.123 | -0.023 |
| Trust towards the state social security system 2011 | -0.083 | 0.135 | 0.033 | -0.142 |
| Lust for life2011 | 0.039 | 0.000 | 0.026 | -0.020 |
| Achieving goals > fun 2011 | -0.076 | -0.094 | -0.074 | 0.151 |
| Success depended on her/himself 2011 | -0.052 | 0.316** | 0.215* | -0.149 |
| Belief in democracy 2011 | 0.071 | -0.039 | 0.191 | 0.026 |
| Supports Law and Justice 2011 | 0.067 | 0.106 | 0.489** | -0.063 |
| Supports no political party 2011 | 0.061 | -0.050 | 0.291*** | 0.162 |
| Fun is most important 2011 | 0.136* | -0.012 | 0.185 | -0.028 |
| Entire life delightful or pleasing 2011 | 0.262*** | 0.039 | -0.144 | -0.048 |
| Money - 1 of 3 most important things in life 2011 | -0.104 | -0.160 | -0.176 | 0.001 |
| No decrease in energy to work 2011 | -0.010 | 0.048 | 0.163 | -0.202 |
| Reforms in Poland successful 2011 | 0.054 | 0.235 | 0.068 | 0.010 |
| Mobile phone 2011 | -0.111 | 0.280 | -0.139 | -0.230 |
| Income 2011 | 0.000 | 0.000 | 0.000 | 0.000*** |
| Health benefits 2011 | 0.168 | 0.066 | 0.122 | 0.102 |
| No wasching machine 2011 | 0.024 | 0.986*** | -0.111 | 0.060 |
| No paid TV 2011 | -0.025 | -0.245 | -0.119 | -0.056 |
| No independent apartment 2011 | -0.173 | -0.648** | -0.639** | -0.386 |
| Size of the living space 2011 | 0.000 | 0.001 | 0.000 | 0.002 |
| Social assistance 2011 | 0.115 | -0.202 | 0.273 | -0.456* |
| Entrepreneur 2011 | -0.344 | 0.405 | -0.966* | -0.695* |
| Self-employed 2011 | -0.217 | 0.201 | -0.199 | -0.497* |
| Unemployed 2011 | 0.125 | -0.333 | -0.505* | 0.266 |
| Inactive (not retired) 2011 | 0.001 | -0.116 | -0.366 | 0.163 |
| Retired (not working) 2011 | -0.264 | 0.125 | - | - |
| Retired working 2011 | -0.169 | -0.302 | - | - |
| Inflexible worktime 2011 | 0.179 | -0.120 | -0.220 | -0.193 |
| Longer breaks in work impossible 2011 | -0.225* | 0.208 | -0.029 | 0.339* |
| Inflexible work (place) 2011 | 0.030 | -0.020 | 0.156 | -0.294 |
| Permanent employment 2011 | 0.012 | 0.206 | -0.347** | 0.035 |
| Working full time 2011 | 0.100 | -0.281 | -0.256 | 0.239 |
| Managers and officials 2011 | -0.558 | 0.161 | 0.922** | 0.336 |
| Professionals 2011 | 0.090 | 0.388 | 0.466 | 0.962** |
| Technicians 2011 | -0.116 | 0.635 | 0.925** | 0.782** |
| Clerical support 2011 | 0.076 | 0.716 | 0.743** | 0.694* |
| Service and sales 2011 | 0.335 | 0.322 | 0.641* | 0.714* |
| Farmers 2011 | 0.103 | 0.103 | -0.075 | 0.176 |
| Craft workers 2011 | 0.024 | 0.016 | 0.608** | 0.444 |
| Plant/machine operators 2011 | -0.019 | 0.225 | 0.330 | 0.463 |
| Elementary occupations 2011 | 0.045 | 0.066 | 0.142 | 0.564 |
| Household ratio of adults sport activity 2011 | 0.242** | -0.031 | 0.346** | 0.252* |
| Household ratio of u20 sport activity 2011 | 0.067 | 0.246 | -0.045 | -0.225 |
| Number of stadiums 2011 | -0.023 | -0.066 | -0.014 | 0.037 |
| Number of fields 2011 | -0.076 | 0.093 | 0.071 | -0.172 |
| Number of indoor arenas 2011 | -0.059 | 0.176 | 0.167 | 0.264* |
| Number of gyms 2011 | -0.001 | 0.160 | 0.118 | -0.184 |
| Number of courts 2011 | -0.068 | -0.020 | -0.103 | -0.135 |
| Number of golf fields 2011 | 0.011 | 0.011 | 0.016 | -0.004 |
| Number of swimming pools 2011 | -0.109 | -0.027 | 0.065 | 0.191 |
| Number of horse tracks 2011 | 0.050* | -0.050 | 0.048 | 0.038 |
| Number of shooting 2011 | 0.002 | -0.009 | -0.069*** | 0.062** |
| Number of winter 2011 | 0.000 | -0.018* | 0.015 | -0.007 |
| Number of motorsports 2011 | 0.021 | -0.009 | -0.034 | -0.049 |
| Number of outdoor 2011 | 0.030 | 0.009 | 0.013 | -0.002 |
| Number of Orliki 2010 | 0.028 | -0.029 | -0.033 | 0.081 |
| Constant | -1.362 | -0.138 | -2.223* | -2.252 |
| Observations | 2,234 | 890 | 933 | 689 |
| Pseudo R-squared | 0.146 | 0.282 | 0.229 | 0.322 |

The table presents results of first-step probit estimation for men stratified by age and past SA. For each stratum, coefficients are reported along with significance, denoted using asterisks: *** p < 0.010, ** p < 0.050, * p < 0.100.
