# Supplementary material for: Impact of sports activity on Polish adults: Self-reported health, social capital & attitudes
Source: PLoS One. 2019 Dec 19;14(12):e0226812. doi: 10.1371/journal.pone.0226812 (PMC6922371; doi:10.1371/journal.pone.0226812)
Supplement: S4 Appendix — (DOCX) [file pone.0226812.s004.docx]

# S4 Appendix. Selective panel attrition.

Following Lechner and Sari (2015) and Pawlowski et al. (2019) we test for selective panel attrition. In other words, for the whole sample for which treatment (SA in 2013) was either applied or not, we test whether remaining in the sample of SD in 2015 was statistically significantly different for treated and non-treated groups. If it was, it might have influenced the results.

Table A presents the results for strata described in the article. In general, there hints of non-random sample selection especially for men – most profoundly, for previously (i.e. in 2011) non-active men (with 0.735 of the 2013 sample remaining in 2015 when initiating SA in 2013 and 0.647 when not; p = 0.002) and previously active men with less than 13 years of education (with 0.721 of these, who continued to be active in 2013 remaining in the sample two years later and 0.621 of these, who ceased it; p = 0.014). Especially for these men, the reported results have to be interpreted with some caution, since SA might have meant, for example, better chances to stay healthy enough to remain in sample two years later (therefore, increasing propensity to be socially active or have a positive attitude as well). Moreover, it might also be an indication of more free time, once again improving the opportunities to engage in social activities. Nevertheless, at least some of the reported results for these strata seem to be stronger than the ones that could result solely from selective sample attrition. In particular, in relative terms, the non-attrition for treated was ca. 16% greater than for the non-treated in both of the highlighted cases, whereas, for example, the average number of declared friends increased by 28% (see Table 4). Furthermore, the beforementioned possible channels of selective attrition are consistent with general interpretation of the reported results.

Table A. Sample attrition test.

| **Stratum** | **Non-active women** | | **Active women** | | **Non-active men** | | **Active men** | |
| --- | --- | --- | --- | --- | --- | --- | --- | --- |
|  | **treatment group** | **difference** | **treatment group** | **difference** | **treatment group** | **difference** | **treatment group** | **difference** |
| Age 41-64 | 0.682 | -0.007 | 0.639 | -0.029 | 0.735 | 0.088*** | 0.677 | 0.036 |
| Age 25-40 | 0.586 | -0.071* | 0.642 | 0.072* | 0.682 | 0.075* | 0.641 | -0.006 |
| High edu level | 0.683 | 0.005 | 0.622 | -0.043 | 0.624 | -0.008 | 0.644 | 0.041 |
| Low edu level | 0.644 | -0.029 | 0.671 | 0.000 | 0.693 | 0.053* | 0.721 | 0.100** |

The table presents results of matching estimation for the 2013 sample stratified by gender, age or education level, and past SA. For each stratum, two values are reported: value of the outcome variable – fraction of the 2013 sample remaining in the 2015 sample - for the treated group (i.e. active in 2013; left column) and estimated difference between the treated group and the non-treated group (i.e. inactive in 2013; right column). For the latter, significance is denoted using asterisks: *** p < 0.010, ** p < 0.050, * p < 0.100.
