# Supplementary material for: Impact of sports activity on Polish adults: Self-reported health, social capital & attitudes
Source: PLoS One. 2019 Dec 19;14(12):e0226812. doi: 10.1371/journal.pone.0226812 (PMC6922371; doi:10.1371/journal.pone.0226812)
Supplement: S5 Appendix — (DOCX) [file pone.0226812.s005.docx]

# S5 Appendix. Additional stratifications.

Apart from results presented in the main part of the article, we have estimated the impact of SA on the same of outcomes but using alternative stratifications – based on area of living (rural vs urban) and marital status, instead of age or education.

Generally, results are comparable – initiation of SA tends to improve social involvement and attitude of women and the area of living is less important in this respect (but particular dimensions differ in this respect). Positive effect on health, however, is stronger for women living in rural areas (Table A).

Table A. Results for women – stratification using area of living.

| **Outcome variable** | **Urban non-active women** | | **Urban active women** | | **Rural non-active women** | | **Rural active women** | |
| --- | --- | --- | --- | --- | --- | --- | --- | --- |
|  | **treatment group** | **difference** | **treatment group** | **difference** | **treatment group** | **difference** | **treatment group** | **difference** |
| *Sport participation* | | | | | | | | |
| Sport 2015 | 0.469 | 0.255*** | 0.772 | 0.34*** | 0.418 | 0.272*** | 0.678 | 0.258*** |
| >1 physical activity 2015 | 0.090 | 0.048** | 0.317 | 0.152*** | 0.089 | 0.073*** | 0.265 | 0.179*** |
| *Health and physical conditions* | | | | | | | | |
| BMI 2015 | 26.466 | 0.010 | 25.495 | -0.044 | 27.067 | 0.191 | 26.375 | -0.086 |
| Dissatisfaction with health 2015 | 3.116 | -0.082 | 2.820 | -0.214** | 2.917 | -0.262*** | 2.96 | 0.086 |
| Physical problems 2015 | 0.699 | -0.008 | 0.607 | -0.025 | 0.696 | -0.033 | 0.699 | 0.060 |
| Health problems 2015 | 0.619 | -0.009 | 0.538 | -0.058 | 0.539 | -0.118*** | 0.617 | 0.017 |
| Seriously ill 2014 | 0.159 | 0.027 | 0.088 | -0.022 | 0.091 | -0.044** | 0.043 | -0.064*** |
| Tiredness not-related to work 2015 | 0.396 | -0.133*** | 0.445 | -0.063 | 0.390 | -0.056 | 0.449 | 0.116** |
| Too much alcohol 2014 | 0.027 | -0.002 | 0.033 | 0.009 | 0.022 | 0.007 | 0.020 | 0.002 |
| *Life attitudes* | | | | | | | | |
| No decrease in energy to work 2015 | 0.708 | 0.106*** | 0.758 | 0.12*** | 0.673 | 0.122*** | 0.673 | 0.035 |
| Entire life delightful or pleasing 2015 | 0.505 | 0.112*** | 0.508 | 0.043 | 0.381 | 0.010 | 0.453 | -0.004 |
| Lust for life 2015 | 8.897 | 0.253** | 8.928 | 0.154 | 8.672 | 0.215* | 8.610 | -0.168 |
| Achieving goals > fun 2015 | 0.653 | -0.004 | 0.668 | 0.017 | 0.640 | 0.013 | 0.599 | -0.077* |
| Success depended on her/himself 2015 | 0.768 | 0.042 | 0.781 | -0.007 | 0.671 | 0.024 | 0.704 | -0.030 |
| Fun is the most important thing 2015 | 0.531 | -0.025 | 0.506 | 0.036 | 0.627 | 0.012 | 0.615 | -0.035 |
| *Social activities* | | | | | | | | |
| Member of sports club 2015 | 0.029 | 0.027*** | 0.005 | 0.005* | 0.000 | - | 0.004 | -0.004 |
| Number of friends met regularly 2015 | 4.600 | 0.256 | 5.775 | 0.69* | 4.77 | 0.576 | 5.782 | 0.063 |
| Number of acquaint. met regularly 2015 | 5.694 | -0.025 | 7.549 | 0.992* | 5.873 | 0.472 | 6.789 | 0.343 |
| Number of friends 2015 | 5.700 | 0.363 | 6.469 | 0.546 | 6.65 | 0.975* | 7.958 | 0.732 |
| Most people can be trusted 2015 | 0.187 | 0.033 | 0.157 | 0.010 | 0.157 | -0.003 | 0.132 | 0.029 |
| Work for local society 2013-2014 | 0.176 | 0.064** | 0.219 | 0.008 | 0.218 | 0.089*** | 0.260 | -0.037 |
| Member of organisations 2015 | 0.260 | 0.096*** | 0.248 | 0.044 | 0.142 | 0.003 | 0.220 | 0.025 |
| Took part in public meeting 2015 | 0.170 | 0.010 | 0.243 | -0.003 | 0.286 | 0.073** | 0.308 | -0.005 |
| Voluntary activities 2015 | 0.114 | 0.051** | 0.098 | 0.009 | 0.087 | 0.033* | 0.136 | 0.042 |
| Voted in elections 2014 | 0.713 | -0.016 | 0.809 | 0.023 | 0.773 | 0.034 | 0.813 | 0.048 |

The table presents results of matching estimation for women stratified by area of living and past SA. For each stratum and each outcome variable, two values are reported: value of the outcome variable for the treated group (i.e. active in 2013; left column) and estimated difference between the treated group and the non-treated group (i.e. inactive in 2013; right column). For the latter, significance is denoted using asterisks: *** p < 0.010, ** p < 0.050, * p < 0.100.

The effects on health, attitude and social involvement is observable (or, at least, more pronounced) in case of married than non-married women, however (Table B). To some extent, this is consistent with stronger impact of SA on women over 40.

Table B. Results for women – stratification using marital status.

| **Outcome variable** | **Non married non-active women** | | **Non married active women** | | **Married non-active women** | | **Married active women** | |
| --- | --- | --- | --- | --- | --- | --- | --- | --- |
|  | **treatment group** | **difference** | **treatment group** | **difference** | **treatment group** | **difference** | **treatment group** | **difference** |
| *Sport participation* | | | | | | | | |
| Sport 2015 | 0.317 | 0.118** | 0.736 | 0.306*** | 0.483 | 0.311*** | 0.750 | 0.326*** |
| >1 physical activity 2015 | 0.096 | 0.062** | 0.271 | 0.105** | 0.089 | 0.060*** | 0.314 | 0.191*** |
| *Health and physical conditions* | | | | | | | | |
| BMI 2015 | 25.953 | -0.504 | 25.346 | -0.263 | 26.903 | 0.175 | 26.204 | -0.019 |
| Dissatisfaction with health 2015 | 3.140 | -0.166 | 2.822 | -0.195 | 2.969 | -0.161** | 2.857 | -0.042 |
| Physical problems 2015 | 0.744 | 0.044 | 0.593 | -0.067 | 0.699 | -0.024 | 0.686 | 0.016 |
| Health problems 2015 | 0.691 | 0.072 | 0.497 | -0.146** | 0.607 | -0.041 | 0.634 | 0.018 |
| Seriously ill 2014 | 0.144 | -0.010 | 0.083 | -0.013 | 0.108 | -0.015 | 0.066 | -0.057*** |
| Tiredness not-related to work 2015 | 0.432 | -0.068 | 0.381 | -0.045 | 0.419 | -0.062** | 0.476 | 0.053 |
| Too much alcohol 2014 | 0.095 | 0.075*** | 0.034 | 0.012 | 0.033 | 0.012 | 0.024 | 0.009 |
| *Life attitiudes* | | | | | | | | |
| No decrease in energy to work 2015 | 0.687 | 0.163*** | 0.733 | 0.044 | 0.689 | 0.096*** | 0.734 | 0.082** |
| Entire life delightful or pleasing 2015 | 0.190 | -0.012 | 0.311 | 0.047 | 0.525 | 0.085*** | 0.561 | 0.006 |
| Lust for life 2015 | 8.575 | 0.226 | 8.602 | -0.033 | 8.884 | 0.275*** | 8.863 | 0.082 |
| Achieving goals > fun 2015 | 0.652 | -0.010 | 0.662 | 0.046 | 0.657 | 0.023 | 0.641 | -0.030 |
| Success depended on her/himself 2015 | 0.692 | 0.019 | 0.708 | -0.049 | 0.732 | 0.039 | 0.759 | 0.011 |
| Fun is the most important thing 2015 | 0.604 | 0.022 | 0.528 | 0.020 | 0.592 | 0.010 | 0.583 | 0.004 |
| *Social activities* | | | | | | | | |
| Member of sports club 2015 | 0.002 | 0.002 | 0.000 | - | 0.006 | 0.005 | 0.005 | 0.002 |
| Number of friends met regularly 2015 | 4.175 | 0.286 | 5.628 | 0.476 | 4.845 | 0.459* | 5.983 | 0.556 |
| Number of acquaint. met regularly 2015 | 5.918 | 0.809 | 7.542 | 0.162 | 5.729 | 0.036 | 7.095 | 0.837 |
| Number of friends 2015 | 5.297 | 0.257 | 6.400 | 0.491 | 6.682 | 1.084*** | 6.76 | 0.184 |
| Most people can be trusted 2015 | 0.159 | 0.012 | 0.176 | 0.026 | 0.203 | 0.044* | 0.138 | 0.011 |
| Work for local society 2013-2014 | 0.099 | -0.002 | 0.191 | -0.146** | 0.201 | 0.074*** | 0.275 | 0.044 |
| Member of organisations 2015 | 0.206 | 0.098* | 0.140 | -0.008 | 0.196 | 0.036 | 0.280 | 0.07* |
| Took part in public meeting 2015 | 0.205 | 0.050 | 0.217 | -0.121** | 0.236 | 0.042* | 0.278 | -0.006 |
| Voluntary activities 2015 | 0.069 | 0.012 | 0.101 | -0.073* | 0.098 | 0.041** | 0.111 | 0.038* |
| Voted in elections 2014 | 0.681 | 0.004 | 0.722 | -0.033 | 0.779 | 0.024 | 0.830 | 0.010 |

The table presents results of matching estimation for women stratified by marital status and past SA. For each stratum and each outcome variable, two values are reported: value of the outcome variable for the treated group (i.e. active in 2013; left column) and estimated difference between the treated group and the non-treated group (i.e. inactive in 2013; right column). For the latter, significance is denoted using asterisks: *** p < 0.010, ** p < 0.050, * p < 0.100.

In case of men, the differences in terms of impact of SA on particular dimensions of social involvement and attitude between inhabitants of urban and rural areas occur (Table C). In general, the effects are less pronounced that for other stratifications, suggesting important heterogeneity not captured by the urban/rural distinction. Positive effects for self-assed health of initiation of SA are, nevertheless, visible. In case of this stratification, as in case of women, the effects of SA on men is stronger for married ones (Table D).

Table C. Results for men – stratification using area of living.

| **Outcome variable** | **Urban non-active men** | | **Urban active men** | | **Rural non-active men** | | **Rural active men** | |
| --- | --- | --- | --- | --- | --- | --- | --- | --- |
|  | **treatment group** | **difference** | **treatment group** | **difference** | **treatment group** | **difference** | **treatment group** | **difference** |
| *Sport participation* | | | | | | | | |
| Sport 2015 | 0.322 | 0.163*** | 0.796 | 0.468*** | 0.308 | 0.186*** | 0.585 | 0.268*** |
| >1 physical activity 2015 | 0.090 | 0.047* | 0.363 | 0.231*** | 0.091 | 0.076*** | 0.208 | 0.176*** |
| *Health and physical conditions* | | | | | | | | |
| BMI 2015 | 27.746 | 0.223 | 27.286 | -0.441 | 27.584 | -0.325 | 27.897 | -0.117 |
| Dissatisfaction with health 2015 | 2.785 | -0.286*** | 2.732 | -0.115 | 2.888 | -0.188** | 2.652 | -0.193* |
| Physical problems 2015 | 0.620 | -0.089** | 0.549 | -0.075 | 0.627 | -0.051 | 0.605 | -0.078 |
| Health problems 2015 | 0.523 | -0.069 | 0.534 | 0.047 | 0.584 | -0.010 | 0.536 | 0.001 |
| Seriously ill 2014 | 0.105 | -.040 | 0.123 | 0.018 | 0.093 | -0.022 | 0.109 | 0.035 |
| Tiredness not-related to work 2015 | 0.372 | -0.027 | 0.358 | 0.032 | 0.410 | 0.050 | 0.347 | 0.034 |
| Too much alcohol 2014 | 0.161 | 0.026 | 0.089 | -0.050 | 0.104 | -0.010 | 0.068 | 0.015 |
| *Life attitiudes* | | | | | | | | |
| No decrease in energy to work 2015 | 0.739 | 0.129*** | 0.769 | 0.037 | 0.635 | 0.024 | 0.778 | 0.094* |
| Entire life delightful or pleasing 2015 | 0.465 | 0.028 | 0.522 | 0.021 | 0.425 | 0.036 | 0.546 | 0.099* |
| Lust for life 2015 | 8.901 | 0.373*** | 8.903 | 0.088 | 8.377 | 0.001 | 8.883 | 0.254 |
| Achieving goals > fun 2015 | 0.474 | -0.104** | 0.549 | -0.101** | 0.582 | 0.037 | 0.651 | 0.118** |
| Success depended on her/himself 2015 | 0.800 | 0.027 | 0.831 | 0.008 | 0.832 | 0.141*** | 0.794 | 0.123** |
| Fun is the most important thing 2015 | 0.745 | 0.149*** | 0.614 | -0.074 | 0.687 | 0.015 | 0.670 | -0.062 |
| *Social activities* | | | | | | | | |
| Member of sports club 2015 | 0.000 | -0.002 | 0.043 | 0.039*** | 0.003 | -0.002 | 0.028 | 0.017 |
| Number of friends met regularly 2015 | 5.360 | 0.721* | 5.667 | -0.463 | 4.710 | 0.196 | 6.508 | 1.734*** |
| Number of acquaint. met regularly 2015 | 7.507 | 2.041*** | 7.888 | 0.095 | 5.987 | -0.222 | 7.766 | 1.917** |
| Number of friends 2015 | 6.386 | 0.805 | 7.068 | 0.253 | 6.802 | 0.858 | 8.267 | 1.376 |
| Most people can be trusted 2015 | 0.163 | 0.050 | 0.192 | 0.001 | 0.147 | 0.004 | 0.146 | 0.001 |
| Work for local society 2013-2014 | 0.153 | 0.041 | 0.236 | -0.007 | 0.202 | 0.051 | 0.308 | 0.079 |
| Member of organisations 2015 | 0.090 | -0.044 | 0.338 | 0.095* | 0.138 | 0.046 | 0.300 | 0.113 |
| Took part in public meeting 2015 | 0.112 | -0.019 | 0.243 | 0.011 | 0.285 | 0.018 | 0.411 | 0.106* |
| Voluntary activities 2015 | 0.059 | 0.009 | 0.097 | -0.087*** | 0.081 | 0.024 | 0.112 | 0.018 |
| Voted in elections 2014 | 0.630 | -0.071* | 0.776 | -0.006 | 0.801 | 0.073** | 0.833 | 0.039 |

The table presents results of matching estimation for men stratified by area of living and past SA. For each stratum and each outcome variable, two values are reported: value of the outcome variable for the treated group (i.e. active in 2013; left column) and estimated difference between the treated group and the non-treated group (i.e. inactive in 2013; right column). For the latter, significance is denoted using asterisks: *** p < 0.010, ** p < 0.050, * p < 0.100.

Table D. Results for men – stratification using marital status.

| **Outcome variable** | **Non married non-active men** | | **Non married active men** | | **Married non-active men** | | **Married active men** | |
| --- | --- | --- | --- | --- | --- | --- | --- | --- |
|  | **treatment group** | **difference** | **treatment group** | **difference** | **treatment group** | **difference** | **treatment group** | **difference** |
| *Sport participation* | | | | | | | | |
| Sport 2015 | 0.427 | 0.284*** | 0.829 | 0.575*** | 0.333 | 0.198*** | 0.724 | 0.365*** |
| >1 physical activity 2015 | 0.110 | 0.087** | 0.338 | 0.168** | 0.085 | 0.057*** | 0.304 | 0.205*** |
| *Health and physical conditions* | | | | | | | | |
| BMI 2015 | 26.698 | 0.036 | 26.598 | 0.392 | 27.981 | -0.114 | 27.817 | -0.429 |
| Dissatisfaction with health 2015 | 2.622 | -0.092 | 2.629 | 0.015 | 2.878 | -0.188** | 2.763 | -0.177** |
| Physical problems 2015 | 0.493 | 0.087 | 0.346 | -0.089 | 0.679 | -0.043 | 0.622 | -0.060 |
| Health problems 2015 | 0.397 | 0.033 | 0.400 | 0.075 | 0.615 | -0.012 | 0.584 | 0.021 |
| Seriously ill 2014 | 0.098 | 0.011 | 0.056 | -0.058 | 0.106 | -0.021 | 0.134 | 0.044* |
| Tiredness not-related to work 2015 | 0.295 | 0.021 | 0.208 | 0.063 | 0.396 | -0.003 | 0.418 | 0.041 |
| Too much alcohol 2014 | 0.111 | -0.016 | 0.104 | 0.000 | 0.132 | 0.021 | 0.099 | 0.026 |
| *Life attitiudes* | | | | | | | | |
| No decrease in energy to work 2015 | 0.730 | 0.013 | 0.795 | -0.125* | 0.674 | 0.073** | 0.747 | 0.067* |
| Entire life delightful or pleasing 2015 | 0.285 | 0.035 | 0.348 | 0.022 | 0.508 | 0.050 | 0.612 | 0.113*** |
| Lust for life 2015 | 8.437 | -0.220 | 8.831 | 0.109 | 8.751 | 0.254** | 8.914 | 0.088 |
| Achieving goals > fun 2015 | 0.362 | -0.083 | 0.487 | -0.098 | 0.569 | -0.017 | 0.595 | -0.010 |
| Success depended on her/himself 2015 | 0.878 | 0.157*** | 0.696 | 0.061 | 0.801 | 0.060** | 0.841 | 0.045 |
| Fun is most important | 0.790 | 0.125** | 0.720 | -0.083 | 0.660 | 0.024 | 0.629 | -0.024 |
| *Social activities* | | | | | | | | |
| Member of sports club 2015 | 0.005 | 0.000 | 0.035 | 0.035 | 0.003 | 0.000 | 0.044 | 0.032** |
| Number of friends met regularly 2015 | 5.253 | 0.838 | 6.709 | 2.245** | 40.831 | 0.264 | 6.093 | 0.520 |
| Number of acquaint. met regularly 2015 | 6.526 | 0.651 | 8.693 | 3.499** | 6.221 | 0.446 | 7.629 | 0.288 |
| Number of friends 2015 | 6.739 | 1.497 | 6.822 | 0.775 | 7.075 | 1.264** | 7.825 | 1.011* |
| Most people can be trusted 2015 | 0.223 | 0.094* | 0.267 | 0.159** | 0.153 | 0.028 | 0.172 | 0.013 |
| Work for local society 2013-2014 | 0.148 | 0.035 | 0.252 | 0.063 | 0.201 | 0.056** | 0.303 | 0.017 |
| Member of organisations 2015 | 0.080 | -0.025 | 0.238 | 0.115 | 0.134 | 0.011 | 0.365 | 0.104** |
| Took part in public meeting 2015 | 0.149 | 0.019 | 0.272 | 0.120 | 0.243 | 0.010 | 0.335 | 0.007 |
| Voluntary activities 2015 | 0.100 | 0.049* | 0.115 | -0.006 | 0.101 | 0.046** | 0.113 | -0.006 |
| Voted in elections 2014 | 0.637 | -0.009 | 0.707 | 0.024 | 0.752 | 0.001 | 0.816 | 0.010 |

The table presents results of matching estimation for men stratified by marital status and past SA. For each stratum and each outcome variable, two values are reported: value of the outcome variable for the treated group (i.e. active in 2013; left column) and estimated difference between the treated group and the non-treated group (i.e. inactive in 2013; right column). For the latter, significance is denoted using asterisks: *** p < 0.010, ** p < 0.050, * p < 0.1.
