# Supplementary material for: Impact of sports activity on Polish adults: Self-reported health, social capital & attitudes
Source: PLoS One. 2019 Dec 19;14(12):e0226812. doi: 10.1371/journal.pone.0226812 (PMC6922371; doi:10.1371/journal.pone.0226812)
Supplement: S6 Appendix — (DOCX) [file pone.0226812.s006.docx]

# S6 Appendix. Estimation results for key outcomes – effects of different technical assumptions.

The ‘Placebo’ effect in Table A presents supposed outcome measured in 2011 as an effect of SA in 2013 (without using the variable in question as an additional balancing variable). Since characteristics in 2011 are supposed to be controlled for, a significant effect would indicate a bias/individual effects not fully controlled for.

Table A. Outcomes of SA – impact of different technical assumptions on difference between treated and non-treated.

|  | **Main result** | **Double weight of propensity score (10)** | **Halved radius (150% of the dist0. to the closest)** | **No bias correction** | **‘Placebo’ effect on corresponding variable in 2011** | **No enforcement of common support** | **Bootstrap (4,999)** | **One person per household** |
| --- | --- | --- | --- | --- | --- | --- | --- | --- |
| *Non-active men 41-64* | | | | | | | | |
| Sport 2015 | 0.197*** | 0.199*** | 0.190*** | 0.231*** | - | 0.179*** | 0.221*** | 0.192*** |
| Dissatisfaction with health 2015 | -0.168* | -0.168* | -0.165* | -0.173** | 0.034 | -0.196** | -0.156* | -0.166* |
| Number of friends 2015 | 1.586*** | 1.604*** | 1.461*** | 1.400*** | -0.126 | 1.690*** | 1.956** | 1.557*** |
| Work for local society 2013-2014 | 0.059** | 0.061** | 0.050* | 0.059** | -0.009 | 0.051* | 0.053 | 0.061** |
| Voluntary activities 2015 | 0.059*** | 0.060*** | 0.062*** | 0.026 | -0.026 | 0.074*** | 0.032 | 0.060*** |
| No decrease in energy to work 2015 | 0.085** | 0.082** | 0.073** | 0.094*** | -0.256 | 0.084** | 0.073 | 0.079** |
| Success depended on her/himself 2015 | 0.100*** | 0.100*** | 0.105*** | 0.096*** | 0.016 | 0.101*** | 0.093** | 0.101*** |
| *Non-active men 25-40* | | | | | | | | |
| Sport 2015 | 0.182*** | 0.186*** | 0.197*** | 0.190*** | - | 0.224*** | - | 0.190*** |
| Dissatisfaction with health 2015 | -0.330*** | -0.330*** | -0.328*** | -0.359*** | -0.127 | -0.346*** | - | -0.339*** |
| Work for local society 2013-2014 | 0.071** | 0.067* | 0.070* | 0.060* | 0.068** | 0.092*** | - | 0.068* |
| *Non-active women 41-64* | | | | | | | | |
| Sport 2015 | 0.302*** | 0.303*** | 0.303*** | 0.283*** | - | 0.328*** | 00.267*** | 0.304*** |
| Dissatisfaction with health 2015 | -0.169** | -0.163** | -0.170** | -0.175** | -0.014 | -0.175** | -0.105 | -0.170** |
| Tiredness non-related to work 2015 | -0.080*** | -0.080*** | -0.086*** | -0.094*** | -0.006 | -0.074** | -0.083** | -0.082*** |
| Number of friends met regularly 2015 | 0.875*** | 0.847*** | 0.948*** | 10.030*** | 0.347 | 0.922*** | 00.747** | 0.898*** |
| Number of friends 2015 | 1.201*** | 1.181*** | 1.038*** | 1.161*** | 0.467 | 1.486*** | 1.030*** | 1.234*** |
| Work for local society 2013-2014 | 0.081*** | 0.079*** | 0.076*** | 0.084*** | 0.022 | 0.080*** | 00.064** | 0.082*** |
| Member of organisations 2015 | 0.078*** | 076*** | 0.080*** | 0.049* | -0.014 | 0.084*** | 0.069** | 0.080*** |
| Took part in a public meeting 2015 | 0.062** | 0.061** | 0.060** | 0.076*** | 0.006 | 0.060** | 0.059* | 0.062** |
| Voluntary activities 2015 | 0.062*** | 0.062*** | 0.067*** | 0.044*** | 0.017 | 0.078*** | 0.051** | 0.063*** |
| No decrease in energy to work 2015 | 0.135*** | 0.135*** | 0.137*** | 0.121*** | -0.015 | 0.164*** | 0.110*** | 0.137*** |
| Lust for life 2015 | 0.289*** | 0.293*** | 0.285*** | 0.378*** | 0.091 | 0.376*** | 0.332*** | 0.300*** |
| *Non-active women 25-40* | | | | | | | | |
| Sport 2015 | 0.242*** | 0.247*** | 0.169*** | 0.203*** | - | 0.207*** | 0.195* | 0.239*** |
| Dissatisfaction with health 2015 | -0.325*** | -0.304*** | -0.249*** | -0.289*** | -0.013 | -0.293*** | -0.233* | -0.338*** |
| Physical problems 2015 | -0.115** | -0.114*** | -0.133*** | -0.116** | -0.007 | -0.177*** | -0.130* | -0.099** |
| Tiredness non-related to work 2015 | -0.117*** | -0.108*** | -0.094** | -0.080* | 0.015 | -0.120*** | -0.070 | -0.118*** |
| Entire life delightful or pleasing 2015 | 0.152*** | 0.151*** | 0.157*** | 0.158*** | -0.036 | 0.079* | 0.142* | 0.178*** |

The table presents results of for the selected key outcomes and strata calculated using alternative technical assumptions. Only the estimated difference between the treated group (i.e. active in 2013) and the non-treated group (i.e. inactive in 2013) is reported, for which significance is denoted using asterisks: *** p < 0.010, ** p < 0.050, * p < 0.100.

For younger, non-active men, the sample size results in most of the boostrap replications being invalid (this problem, but to lesser extent also takes place for younger women).

Furthermore, to test whether possibility of inclusion of more than one person from a given household might violate the results, estimation procedure was also conducted on smaller subsample – constructed by choosing randomly only one respondent participating the individual survey of a given strata from each household (if there was at least one such a person).
